# Engineering *Escherichia coli* for polyethylene terephthalate powder biodegradation via recoding of an outer membrane protein

**DOI:** 10.1016/j.isci.2025.114621

**Published:** 2026-01-02

**Authors:** Joan Giménez-Dejoz, Paula Vidal, Sonia Romero, David Almendral, Miguel Luengo, Mireia Martinez-Sugrañes, Jose L. Gonzalez-Alfonso, Ana Robles-Martín, Francisco J. Plou, Rafael Bargiela, Martin Floor, Manuel Ferrer, Víctor Guallar, Laura Fernandez-Lopez

**Affiliations:** 1 Barcelona Supercomputing Center (BSC), 08034 Barcelona, Spain; 2 Instituto de Catalisis y Petroleoquimica (ICP), CSIC, 28049 Madrid, Spain; 3 PhD program in Biotechnology, Faculty of Pharmacy and Food Sciences, University of Barcelona, 08028 Barcelona, Spain; 4 Institució Catalana de Recerca i Estudis Avançats (ICREA), 08010 Barcelona, Spain

**Keywords:** Computational molecular modeling, Molecular dynamics, Biological sciences, Protein, Bioengineering, Metabolic engineering, Synthetic biology

## Abstract

Deep computationally guided protein design enabled the introduction of a Ser-His-Asp catalytic triad that supports polyethylene terephthalate (PET) hydrolysis within an inner membrane *Escherichia coli* protein. This allows the engineering, through gene editing, of a strain capable of degrading PET particles smaller than 4.5 nm. We extended this approach to PET powder (<300 μm) using a computational workflow that builds geometrically pre-organized catalytic triads while preserving substrate binding in extracellular or surface-exposed membrane proteins. Four additional proteins were reprogrammed to degrade PET. Replacement of the outer membrane protein OmpA with a PETase-active variant carrying a surface-exposed artificial triad enabled an engineered strain to release 157 ± 2 μM hydrolysis products within 24 h at 37°C and to sustain growth (0.18 ± 0.07 h^−1^) on PET powder as a carbon source. These results demonstrate the feasibility of engineering *E*. *coli* strains for PET powder biodegradation without exogenous PETase genes.

## Highlights

• *Triad exploration* designs Ser-His-Asp/Glu catalytic triads across protein surfaces
• Four secreted or surface-exposed proteins were recoded for PET hydrolysis
• OmpA gene editing added a surface triad enabling PET powder hydrolysis by *E*. *coli*
• Engineered strain released 157 μM products and grew (0.18 h^−1^) on PET powder

## Graphical abstract

**Figure:**
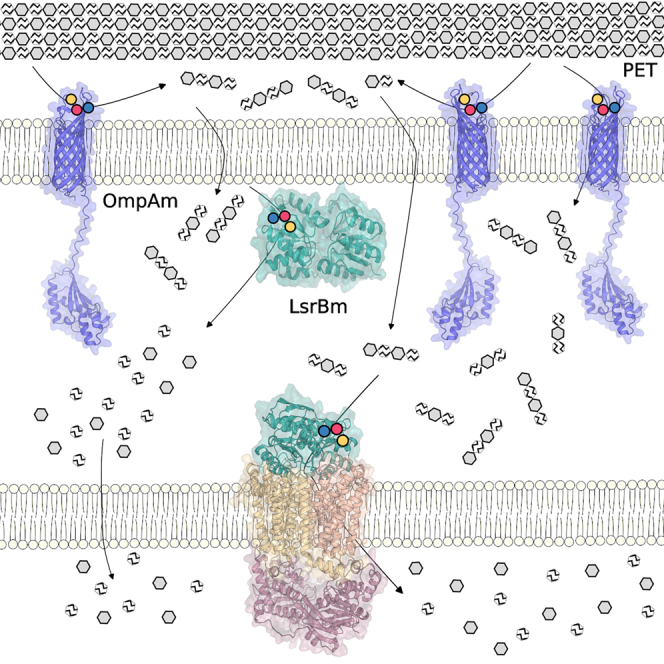

## Introduction

Without intervention, plastic production, which is currently 400 million tons (Mt) year^−1^, will increase by 37% by 2050, accompanied by declining recycling rates and a doubling of mismanaged plastics. However, a combination of research and policy measures could reduce mismanaged plastic waste by up to 91%,^1^^,^^2^ together with plastic-related greenhouse gas emissions by 33%. This reduction is urgently needed because plastics, such as macroplastics (>5 mm) and increasingly relevant microplastics (<5 mm) and nanoplastics (<1 μm), inevitably accumulate in vast quantities in the environment. For example, globally, the estimated amount of macroplastic waste is 400 Mt per year,^3^ and the global emissions of micro and nanoplastics are estimated at 10–40 Mt per year.^4^ This urgency is further underscored by the fact that the total amount of plastic ever produced (4,977 Mt) is projected to reach 12,000 Mt by 2050;^5^ however, only approximately 9% is recycled effectively.^6^ This low percentage is largely because, until recently, the biodegradation and enzymatic recycling of hydrolyzable plastics, particularly polyethylene terephthalate (PET), had not been considered economically viable alternatives. This perception has shifted as the cost of enzymatic recycling using PET-degrading enzymes (PETases) has decreased to approximately 4% of the market price of virgin PET per ton (US $25 kg^−1^ of protein).^7^^,^^8^ To achieve a PET-based plastic bioeconomy, a combined effort involving enzyme and microbial strain discovery and engineering, along with process optimization and standardization, is needed.^9^^,^^10^^,^^11^

To date, multiple and widely distributed types of organisms have been identified as naturally possessing PET-degrading machinery, from which enzymes potentially capable of degrading PET-type plastics have been cataloged.^12^^,^^13^ For example, a recent global assessment detected approximately 1,600 *Ideonella sakaiensis* PETase (*Is*PETase)-like genes in 31 marine surface samples and throughout the water column, including the deep sea, underscoring their widespread presence in marine ecosystems.^14^ These findings were further supported by a search across 83 million nonredundant (NR) gene clusters from 415 ocean samples, revealing 75 *Is*PETase-like candidates.^15^ In addition, a comprehensive sequence search in the National Center for Biotechnology Information (NCBI) NR protein sequence database revealed a vast repertoire of 33,247,501 candidates, which was reduced to 436,488 after clustering at 50% sequence identity, potentially encompassing nearly all PETase-like sequences with structural similarity to known PETases.^16^ The identification of these and other PETases has been facilitated by advances in bioinformatics, structure- and function-informed algorithms, machine learning and artificial intelligence methods, and deep learning protein language models that accelerate enzyme exploration.^15^^,^^16^^,^^17^^,^^18^

However, native PETases are not fully optimized for PET degradation under industrially relevant conditions,^19^ and this feature has motivated the development of engineered enzymes with catalytic performances approaching those required for effective plastic waste biodegradation and recycling. Notable examples include specialized benchmark variants such as LCC-ICCG,^7^ HotPETase,^20^ and TurboPETase.^19^ The design of these variants has been supported by molecular-level studies using quantum mechanics/molecular mechanics simulations to elucidate the chemical reaction mechanisms of PET-degrading enzymes,^21^^,^^22^ along with computational redesign strategies.^19^ Such mechanistic insights are critical for guiding the development of enzyme variants with high performance under conditions relevant to large-scale hydrolysis.^23^

The availability of PETases, either naturally occurring or engineered, has enabled synthetic biology and metabolic engineering strategies to endow microorganisms that naturally lack PET-degrading capabilities with this function. These strategies rely, for example, on plasmid-based expression systems or genome *knock*-*in* via CRISPR or homologous recombination to produce exogenous PETases (native, engineered, or synthetic chimeric proteins), either secreted or anchored to the cell surface, thereby generating engineered microbes capable of PET biodegradation (Table S1). If additional gene sets are incorporated, these microbes can also perform PET upcycling.^24^ One recent innovation is the implementation of GenRewire, a genome-rewiring strategy that combines structure-guided protein engineering with CRISPR-Cas9 editing to repurpose endogenous proteins for PET-degrading activity, thereby enabling microorganisms that naturally lack this capacity to degrade PET without the need for exogenous PETase genes.^25^ This method combines protein energy landscape exploration (PELE)-based simulations using soluble PET oligomers, the insertion of Ser-His-Asp catalytic triads with potential ester-hydrolyzing activity, and CRISPR-mediated genome editing to replace native proteins with PETase-active variants. Using this approach, we successfully introduced artificial catalytic triads into three proteins from *E*. *coli* BL21 (DE3), demonstrating that endogenous *E*. *coli* proteins can be repurposed for PET degradation without affecting their native function; however, their PETase activity remains lower than that of benchmark PETases such as LCC-ICCG and HotPETase.^25^ Furthermore, we demonstrated that *E*. *coli* BL21 (DE3) can be engineered to degrade PET nanoparticles (nPET, <5 nm, 1.3% crystallinity) without the introduction of exogenous PETase genetic material but rather by replacing one of its own proteins, an inner membrane protein named LsrB, with a variant repurposed for PET degradation via CRISPR-Cas9.^25^ However, the periplasmic localization of LsrB restricted access to larger PET substrates such as PET powder (pPET, <300 μm, >40% crystallinity), limiting its applicability to nPET degradation.

To overcome this constraint and facilitate direct interaction with extracellular PET, we optimized our computational workflow, transitioning from a substrate-centered *ligand exploration* protocol to a triad-first *triad exploration* strategy, which systematically identifies geometrically pre-organized catalytic triads while preserving substrate binding, and targeted *E*. *coli* proteins predicted to be secreted or localized in the outer membrane. Once repurposed with catalytic triads and genomically integrated, we evaluated whether the newly repurposed proteins conferred to *E*. *coli* the ability to degrade PET particles (pPET, <300 μm, >40% crystallinity) at mesophilic temperatures and to utilize it as a carbon source for growth. Overall, the findings expand the repertoire of PETases and underscore the potential of genome-rewiring strategies as powerful tools for constructing programmable microbial systems for PET biodegradation.

## Results

### Advancing catalytic triad design for protein repurposing

GenRewire proved feasible for introducing Ser-His-Asp catalytic triads into regions of *E*. *coli* proteins predicted to bind PET-derived oligomers, with a significant 38% hit rate for successfully acquiring PETase activity.^25^ In this initial proof of concept, we applied the *ligand exploration* protocol, where we sought to identify potential substrate binding sites through extensive PELE simulations. In this approach, the ligand freely explores the protein surface to identify favorable binding pockets, which is a computationally demanding task. We used three soluble PET-derived products as ligands, ETETETE, ETETE, and TE, where T and E represent terephthalic acid and ethylene glycol (EG), respectively.^26^ Afterward, candidate positions for introducing a catalytic triad were evaluated near the identified binding site. Although this method was shown to be effective in locating high-affinity ligand-protein interactions and in creating new catalytic sites,^25^^,^^27^^,^^28^^,^^29^^,^^30^ it is limited to inserting catalytic triads near the identified binding sites, which risks altering the binding site architecture and negatively affecting substrate affinity. Moreover, because we can diffuse only relatively small ligands, the approach tends to locate partly buried pockets, which might hinder the binding of larger oligomers, such as crystalline PET.

**Figure 1:**
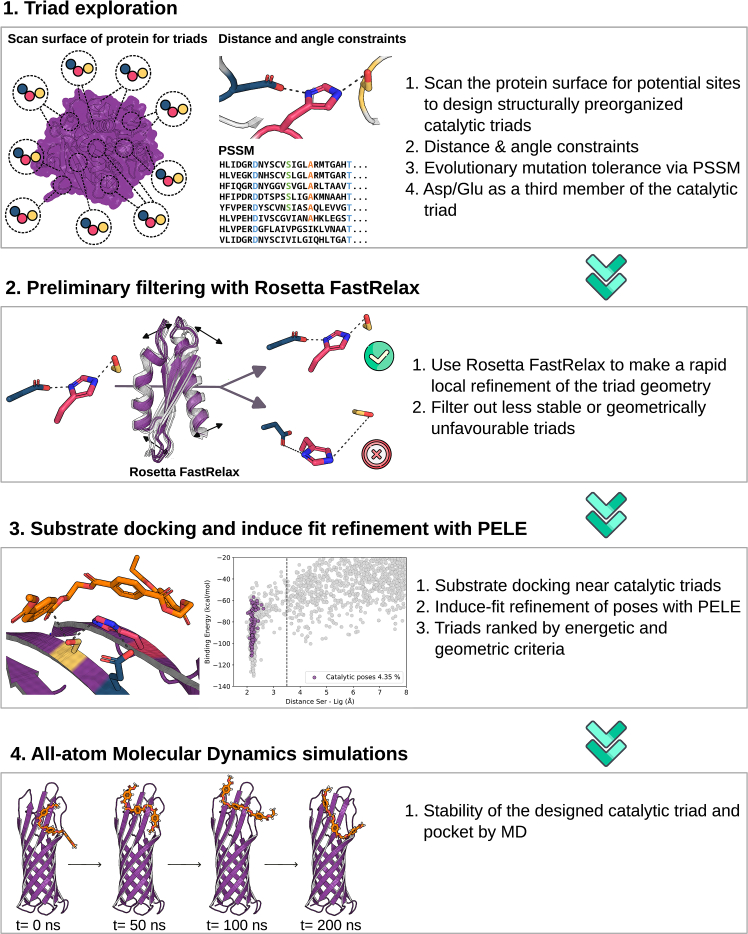
Schematic representation of the computational *triad exploration* workflow for catalytic triad exploration and design Step 1: the process begins with an exploration of the protein surface to design potential triads on the basis of geometry, distance, and angular constraints while minimizing structural disturbance using precomputed PSSMs, considering both aspartic acid (Asp) and glutamic acid (Glu) for the third residue. Step 2: unfavorable designs are rapidly filtered out using the Rosetta FastRelax protocol to ensure triad preorganization and structural stability. Step 3: the substrate is docked near the preorganized catalytic triad. This is followed by refinement and filtering using a PELE to identify the best catalytic poses that maintain the distances crucial for the reaction mechanism. Step 4: the top-ranked designs undergo all-atom MD simulations for final validation of the substrate binding mode and the stability and dynamics of the engineered catalytic triad.

To overcome these limitations, we developed an improved version of our workflow focused on catalytic triad design that we named *triad exploration* (Figure 1). This protocol inverts the logic of the previous approach. Specifically, rather than first locating the substrate binding site and then designing a catalytic triad around it,^25^^,^^31^ we search on the protein surface for all potential sites where we can systematically design structurally preorganized catalytic triads. Only after engineering viable triads did we search for compatible substrate binding sites in their vicinity. An advantage of this shift in our approach is that it inherently preserves the substrate-binding site, reducing the risk of disrupting substrate binding interactions upon subsequent triad insertion. Moreover, the number of possible catalytic triads is typically much greater than that in the previous protocol, as it is not limited to the identified substrate binding site in the PELE via the global exploration (GE) module (PELE GE).

To enhance the reliability and efficiency of this triad-first approach, we also introduced several methodological improvements on the basis of insights from our previous work.^25^ First, in addition to the traditional hydrogen bond distance criteria for triad formation, we incorporated angular constraints to ensure proper geometric orientation for catalysis. These constraints were derived from statistical analysis of serine hydrolase structures in the Protein DataBank and included an optimal catalytic angle range of 100°–180°, representing the geometrical configuration most compatible with productive catalysis in serine hydrolases, ensuring proper alignment of the Ser-His-Asp/Glu triad,^32^ and supported by a retrospective analysis of previous PluriZymes in which similar triads had been successfully incorporated into different scaffolds (see STAR Methods).^25^^,^^27^^,^^28^^,^^29^^,^^30^ Second, we expanded the design space by including glutamic acid (Glu) alongside aspartic acid (Asp) as a potential third member of the catalytic triad of typical ester hydrolases,^33^ increasing the likelihood of satisfying the geometric and distance requirements. Third, we addressed the potential protein structural disruptions caused by the introduction of few precise amino acid changes. Using position-specific scoring matrices (PSSMs), we evaluated the evolutionary tolerance of each candidate substitution site, enabling us to avoid changes at highly conserved positions that might compromise protein folding or stability. By integrating evolutionary constraints into the design pipeline, we aimed to preserve protein integrity while enhancing functional potential. Finally, to further reduce the computational cost, we included a preliminary filtering step using the Rosetta *FastRelax* protocol. This allows for rapid local refinement of the triad geometry, enabling early elimination of poor candidates before engaging in more intensive PELE or molecular dynamics (MDs) simulations.

### Computational selection and catalytic triad engineering of outer membrane proteins

We applied our *triad exploration* protocol to 43 *E*. *coli* proteins (Table S2), which, in accordance with the EchoLOCATION and STEP databases and proteomic experimental studies,^34^^,^^35^^,^^36^ included outer membrane β-barrel proteins, secreted proteins, and cell surface-associated proteins. Focusing on proteins with these localizations ensured the possibility that engineered variants with PETase activity could access PET particles of any size. To further validate the method, we also tested periplasmic and cytoplasmic proteins, thereby covering a broad range of subcellular localizations.

Notably, among these forty-three proteins, nineteen (Table S2) had already been evaluated with the previous version of GenRewire.^25^ In that study, the protocol was restricted to searching for catalytic triads near the predicted substrate-binding site (using ETETETE, ETETE, or TE as ligands). In contrast, in the present work, we scanned the entire protein surface, enabling the identification of additional triads not detected in the initial analysis. These triads were filtered on the basis of strict distance and angular constraints (see STAR Methods), ensuring a pre-organized geometry consistent with that of known serine hydrolases. To minimize structural disruption and improve the likelihood of stable expression, we generated PSSMs for each protein and targeted only substitution sites for the triad residues with favorable evolutionary propensity profiles. We also excluded triads located within 6 Å of known native catalytic residues to avoid interference with wild-type functions. This initial triad screening phase yielded 15,757 possible variants. To assess the pre-organization and structural viability of each design, we applied the Rosetta *FastRelax* protocol, performing 30 independent relaxation trajectories per model and measuring the inter-residue distances within the catalytic triad. Variants were retained only if at least 50% of relaxed structures preserved the catalytically competent triad geometry, reducing the pool to 397 candidates.

After triad design and filtering, each variant was subjected to substrate docking using a soluble product that is part of PET as a substrate model, namely, ETETETE.^26^ Docking was guided by spatial constraints, which restricted ligand placement within 5 Å of the hydroxyl oxygen of the catalytic serine residue to ensure proximity for potential catalysis. From the resulting poses, we selected the best-scoring configuration for each variant on the basis of the docking score, serine-ligand distance, and optimal arrangement of the catalytic triad residues. These representative complexes were then evaluated using two rounds of PELE simulations to explore induced fit dynamics and estimate catalytic performance. To quantitatively rank each variant, we used the free energy catalytic matrix (see STAR Methods), which integrates energetic and geometric criteria relative to those of reference PETases. Following the first PELE round, 133 variants were selected for further refinement. Finally, the 59 top candidates were advanced to all-atom MD simulations for a more detailed assessment of catalytic geometry stability over time. We applied a final selection step based on three additional structural and evolutionary criteria to prioritize candidates for experimental validation. First, we evaluated the stability of the designed catalytic triad throughout the MD simulations. Variants were retained only if they maintained catalytically competent triad geometries in at least 37% of the simulation frames, ensuring both a reasonable likelihood of sustained activity and a manageable number of sequences for experimental validation. Second, we examined the proximity of residues capable of acting as an oxyanion hole near the catalytic serine, a key feature for stabilizing reaction intermediates in serine hydrolases. Finally, we evaluated the solvent accessibility of the catalytic serine to ensure that it was not buried within the protein core,^37^ and only those variants with adequately accessible serine residues were considered suitable.

**Figure 2:**
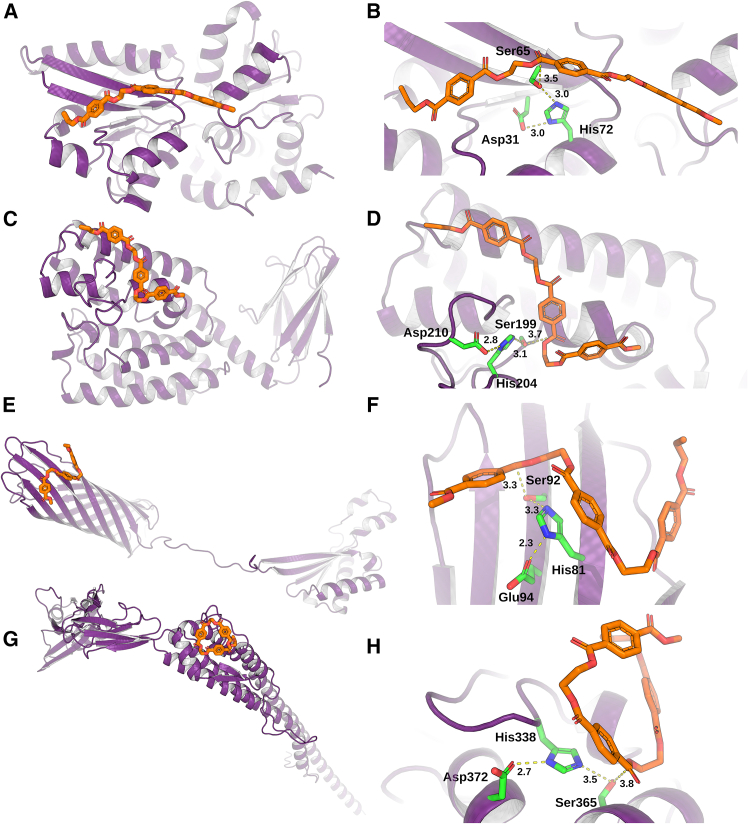
Structures and active-site architectures of engineered *E*. *coli* proteins with PETase activity (A, C, E, and G) Structures of the four protein variants, including CysP_m_ (A), EfeO_m_ (C), OmpA_m_ (E), and FliD_m_ (G), successfully produced in soluble form and displaying PETase activity. Structures of the corresponding recoded proteins with the PET oligomer substrate ETETETE bound at the engineered active sites are shown. UniProt identifiers are provided in Table S3. Structural models of CysP, EfeO, OmpA, and FliD were obtained from the AlphaFold database (AF-P16700-F1-v6, AF-P0AB24-F1-v6, AF-P0A910-F1-v6, and AF-P24216-F1-v6, respectively; for details, see STAR Methods). The structures of the proteins, shown as monomers, and the ligands were extracted from the PELE poses. However, their native oligomeric states are CysP_m_ (monomer), EfeO_m_ (dimer), OmpA_m_ (monomer, although it can form a dimer in the outer membrane under specific conditions), and FliD_m_ (hexamer). (B, D, F, and H) Details of the engineered active sites, corresponding to the protein variants shown in (A, C, E, and G), respectively, and illustrating the introduced serine-histidine-aspartate/glutamate catalytic triads and the positioning of the PET substrate (ETETETE). (A–H) Proteins are shown as purple cartoons, the PET substrate as orange sticks, and the engineered catalytic triad as green sticks, with the key catalytic contacts and distances highlighted by dashed yellow lines.

The application of this workflow yielded 12 *E*. *coli* BL21 (DE3) variants from 11 proteins, including integral membrane, membrane-anchored, surface appendage, and periplasmic proteins with membrane anchors. These proteins were repurposed for presumptive PETase activity and, when produced in soluble form, could be tested to validate such activity. The structures of the variants found to be active, along with their database protein names and identifiers, and the specific residues forming the serine-histidine-aspartate/glutamate triads, are shown in Figure 2. See also Table S3 for complete information, including the residue substitutions introduced to generate the serine-histidine-aspartate/glutamate triads in all variants repurposed as presumptive PETases.

### *In vitro* evaluation of PETase activity in engineered *E*. *coli* proteins

Eleven synthetic wild-type proteins and the 12 corresponding variants, all N-terminal hexahistidine-tagged, were successfully produced in soluble form after heterologous expression in *E*. *coli* BL21 (DE3) using the pET-45b(+) vector and were subsequently purified using affinity chromatography with HIS-Select Nickel Affinity Gel (STAR Methods) (Figures S1 and S2). The purification yields ranged from 1.2 to 138.2 mg per liter of culture.

We first evaluated the ability of the 12 variants to hydrolyze commercial PET (maximum particle size, 300 μm; crystallinity, >40%) and nPET prepared from PET film (average particle size, 4.5 nm [range, 1.0–5.5 nm]; crystallinity, 1.3%).^25^ The assays were performed as detailed in Table 1 at pH 7.0°C and 37°C, as well as at 40°C and 60°C for up to 24 h (Figure S3 and Table S4). These standardized conditions allowed reproducible comparisons of the hydrolytic activities of engineered variants with those of previously reported designs and benchmark PETases;^25^ however, they differ from conditions relevant to large-scale hydrolysis.^23^ Degradation products were identified by ultrahigh-performance liquid chromatography (UHPLC) with electrospray ionization-mass spectrometry (Figure S4) and quantified with high-performance liquid chromatography (HPLC) coupled with a photodiode array detector^25^ (see STAR Methods).

**Table 1:** Specific activities of engineered variants toward pPET and nPET compared to those of previously reported similar designs and optimized benchmark PETase variants

Protein ID	Amino acid substitutions	Artificial active site	ETE hydrolysis at 37°C (μmol min^−1^ g^−1^)^a^	PET hydrolysis at 37°C (μmol h^−1^ g^−1^)^a^	
				nPET	pPET
CysP_m_	Q72H, S31D	S65, H72, D31	61 ± 0.2∗	74 ± 5	8 ± 2
OmpA_m_	A92S, R81H, K94E	S92, H81, E94	2,738 ± 7∗∗	222 ± 18	36 ± 1
EfeO_m_	A199S, Y204H	S199, H204, D210	57 ± 0.0∗	0 (750 ± 10^b^)	0 (20 ± 1^b^)
FliD_m_	N260S, E240H, N253E	S365, H338, D372	9 ± 0.4∗	52 ± 5	0 (100 ± 1^b^)
SsuA_m_^c^	A65S, P67H, P87D	S65, H67, D87	369 ± 8	2,160 ± 40	220 ± 0
LsrB_m_^c^	G318S, D317H, S287D	S318, H317, D287	503 ± 8	5,150 ± 90	860 ± 100
HotPETase^c^	–	–	–	10,360 ± 99	1,750 ± 170
LCC-ICCG^c^	–	–	–	12,600 ± 115	1,900 ± 80

a Reaction conditions, [ETE], 2 mM, [pPET], 7 mg mL^−1^ or [nPET], 1.65 mg mL^−1^; [enzyme], 0.1 mg mL^−1^; buffer, 50 μL of 40 mM 4-(2-hydroxyethyl)-1-piperazineethanesulfonic acid (HEPES) buffer; pH, 7.0; agitation, 950 rpm. The reaction times were set to 0, 2, 4 and 24 h (only 24 h data were used for calculations, unless noted with ∗ for 4 h and ∗∗ for 5 min). Analyses were performed by HPLC. All values represent the means of three independent biological replicates (*n* = 3), with standard deviations calculated using the STDEV. The S function in Excel 2024 was used. pPET was obtained from GoodFellow Cambridge (Huntingdon, UK; ref. ES30PD000132; maximum particle size: 300 μm; crystallinity: >40%). nPET was prepared from amorphous PET (GoodFellow Cambridge, Huntingdon, UK; ref. ES301445) (average particle size: 4.5 nm [range, 1.0–5.5 nm], crystallinity: 1.3%). Unprocessed data in Data S1A–S1C and unprocessed HPLC data and MS files in Data S2A and S2B.

b Values in brackets refer to the values obtained at 60°C, as no activity was detected at 37°C.

c Values from Vidal et al.^25^ Reaction conditions, [pPET], 7 mg mL^−1^ or [nPET], 1.65 mg mL^−1^; [enzyme], 0.1 mg mL^−1^; buffer, 50 μL of 40 mM HEPES buffer; pH, 7.0; agitation, 950 rpm.

The results demonstrated that two amino acid changes in CysP (Q72H, S31D), a sulfate-binding component of the ABC-type sulfate transporter system,^38^ and three in OmpA (A92S, R81H, K94E), a pore-forming outer membrane protein,^39^ successfully generated functional Ser-Asp/Glu-His catalytic triads (CysP_m_: Ser65-Asp31-His72; OmpA_m_: Ser92-Glu94-His81), as both CysP_m_ and OmpA_m_ were able to degrade nPET and pPET (Table 1). At 37°C, the hydrolytic activities were 74 ± 5 and 222 ± 18 μmol h^−1^ g^−1^ on nPET and 8 ± 2 and 36 ± 1 μmol h^−1^ g^−1^ on pPET for CysP_m_ and OmpA_m_, respectively (Table 1). Both variants also retained activity at 40°C and showed significantly higher activity at 60°C (Table S4). Two amino acid changes in EfeO (A199S and Y204H), an integral membrane iron-permease component of an iron uptake system,^40^ and three in FliD (N260S, E240H, and N253E), a flagellar hook-associated protein,^41^ also generated functional Ser-His-Asp catalytic triads (EfeO_m_: Ser199-Asp210-His204; FliD_m_: Ser365-Asp372-His338), supporting PET hydrolysis (Table 1). At 37°C, no detectable activity was observed for EfeO_m_ on either substrate, whereas activity was recovered at 60°C (750 ± 10 μmol h^−1^ g^−1^ on nPET and 20 ± 1 μmol h^−1^ g^−1^ on pPET). In contrast, FliD_m_ hydrolyzed nPET at 37°C (52 ± 5 μmol h^−1^ g^−1^) but did not affect pPET under these conditions, with activity detected only at 60°C (100 ± 1 μmol h^−1^ g^−1^) (Table S4). Unlike CysP_m_ and OmpA_m_, these engineered variants exhibited PETase activity on pPET only at elevated temperatures. Using HPLC with well-characterized standards,^25^ in conjunction with UHPLC-MS, we confirmed that the degradation products included ETE, TE, and T, as well as several other oligomers (referred to as “others”), such as TET and TETE (Figure S4), depending on the engineered variant. Their concentrations varied depending on the engineered variant and assay conditions (temperatures of 37, 40, or 60°C; see Figure S3). The different degradation products identified for each *E*. *coli* BL21(DE3) protein engineered to display PETase activity are listed in Table S5.

The hydrolytic activity of the PET-degrading engineered variants was also assessed using the soluble PET oligomer ETE as a substrate (Table 1). At 37°C, the optimal growth temperature of *E*. *coli*, OmpA_m_ exhibited the highest activity among the engineered proteins, reaching 2,738 ± 7 μmol min^−1^ g^−1^. This value is higher than that of the other *E*. *coli* proteins repurposed with the same methodology, namely, LsrB_m_ (503 ± 8 μmol min^−1^ g^−1^) and SsuA_m_ (369 ± 8 μmol min^−1^ g^−1^). On the other hand, only CysP_m_, EfeO_m_, and FliD_m_ exhibited modest activity (61 ± 0.2, 57 ± 0.0, and 9 ± 0.4 μmol min^−1^ g^−1^, respectively).

**Figure 3:**
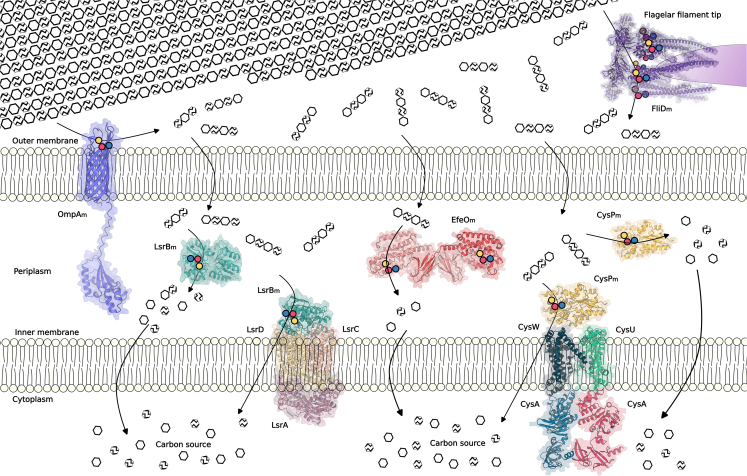
Subcellular localization of *E*. *coli* BL21 (DE3) proteins repurposed to acquire PETase activity on the basis of the EchoLOCATION and STEP databases Schematic representation of the *E*. *coli* cell envelope, comprising inner and outer membranes, with proteins repurposed as PETases (OmpA_m_, EfeO_m_, CysP_m_, FliD_m_, and previously engineered LsrB_m_^25^) shown in their predicted conformations and localizations. The polymeric substrate, crystalline PET, is depicted as hexagonal units, where ethylene glycol moieties are represented by solid hexamers and terephthalic acid moieties by striped hexamers. The schematic diagram highlights the spatial arrangement of the proteins within the cellular context and the chemical products generated during PET hydrolysis. Designed catalytic triads in each protein are indicated as yellow, blue, and red spheres.

To contextualize their performance, the activities of OmpA_m_, CysP_m_, EfeO_m_, and FliD_m_ were compared with those of other *E*. *coli* proteins repurposed using the same methodology, namely, LsrB_m_ and SsuA_m_, and with benchmark-engineered PETases (Table 1), all under the same reaction conditions. LsrB_m_ and SsuA_m_ displayed markedly higher activities, reaching 5,150 ± 90 and 2,160 ± 40 μmol h^−1^ g^−1^ on nPET and 860 ± 100 and 220 ± 0 μmol h^−1^ g^−1^ on pPET, respectively, at 37°C. Benchmark PETases such as HotPETase and LCC-ICCG also outperformed the repurposed variants on pPET, with activities of 1,750 ± 170 and 1,900 ± 80 μmol h^−1^ g^−1^, respectively, at 37°C. These comparisons highlight that although the newly repurposed proteins exhibited modest hydrolytic efficiency, they nevertheless represent a new set of endogenous *E*. *coli* membrane-associated and periplasmic proteins (Figure 3) that were successfully reprogrammed to acquire PET-degrading activity.

### Genome rewiring enables *E*. *coli*-mediated biodegradation of PET powder

To generate a genome-rewired *E*. *coli* strain capable of depolymerizing pPET with a maximum particle size of 300 μm and a crystallinity >40%, we replaced *ompA*_WT_ with *ompA*_m_ in the *E*. *coli* BL21 (DE3) genome using CRISPR-Cas9 (see STAR Methods). We selected OmpA for replacement rather than any of the other three proteins studied here because OmpA_m_ showed the best performance in hydrolyzing not only pPET but also nPET and ETE at 37°C (Table 1). Instead of using the parental *E*. *coli* BL21 (DE3) strain as a host, we employed the strain *E*. *coli* BL21 (DE3) Δ*lsrB*:*lsrB*_m_/pET-45b(+) (*aldA*, *fucO*], which was constructed previously.^25^ This strain, referred to as strain S1, is capable of degrading nPET through the activity of LsrB_m_ but not pPET and supports safe biomass production via AldA and FucO, which channel EG into central metabolism through the glycerate pathway.^42^ Thus, the resulting strain was able to degrade nPET and use the released products for growth. After *ompA*_*WT*_ was replaced with *ompA*_*m*_, the resulting strain, *E*. *coli* BL21 (DE3) Δ*lsrB* Δ*ompA*:*lsrB*_*m*_ *ompA*_*m*_/pET-45b(+) [*aldA*, *fucO*], herein referred to as strain S3, was constructed. In this way, strain S3 carries *lsrB*_m_ and *ompA*_m_ integrated into the genome, whereas *aldA* and *fucO* are incorporated heterologously.

**Figure 4:**
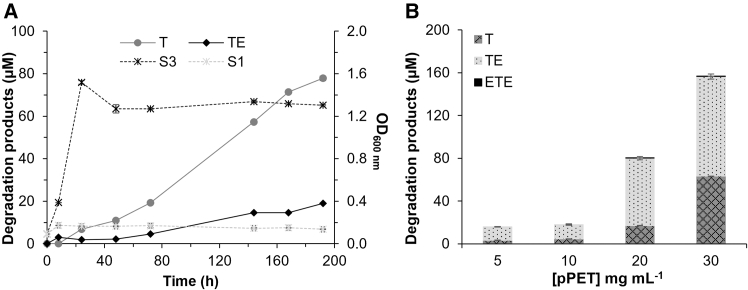
*In vivo* accumulation of PET degradation products by genome-rewired *E*. *coli* cultured with PET powder (A) Concentrations of degradation products generated during *in vivo* cultivation of strain S3 in the presence of pPET (solid lines, primary axis) and the growth of strains S3 and control S1 during this experiment (dotted lines, secondary axis). Cultures (10 mL) were grown in 50 mL Falcon tubes for 192 h in M9 medium supplemented with 0.1 mg mL^−1^ ampicillin at 37°C and 200 rpm, with an initial OD_600 nm_ of 0.1 and a pPET concentration of 5.0 mg mL^−1^. To induce growth via EG utilization, 1 mM isopropyl β-D-1-thiogalactopyranoside (IPTG) and 0.1 mg mL^−1^ glycerol were added. We would like to note that in our previous work,^25^ strains expressing only the recoded PETase-like protein (LsrB_m_) or only the EG-metabolizing genes did not grow, confirming their inability to use EG as a carbon source. These controls were therefore not repeated here; instead, strain S1, which carries both heterologous EG-metabolizing genes and the recoded periplasmic LsrB_m_, was used as the main control, as it can hydrolyze nanometric PET particles but not PET powder, as shown in this study. (B) Concentrations of degradation products generated during *in vivo* cultivation of strain S3 in the presence of different concentrations of pPET. Reactions were performed in 2-mL Eppendorf tubes using 500 μL of culture (OD_600 nm_ of 0.1) in M9 medium supplemented with 0.1 mg mL^−1^ ampicillin, 1 mM IPTG, 0.1 mg mL^−1^ glycerol, and different concentrations of pPET (5, 10, 20, and 30 mg mL^−1^) at 37°C and 1,200 rpm. (A and B) Absorbance in (A) was quantified spectrophotometrically, and the degradation products in (A and B) were quantified using HPLC, as detailed in STAR Methods. The values represent the means of three biological replicates (*n* = 3), with the means and standard deviations (SDs) shown. Unprocessed data are available in Data S1D and S1F.

Unlike LsrB_m_, which is located in the inner membrane, OmpA_m_ forms a pore in the outer membrane, with its engineered catalytic center exposed on the cell surface, potentially enabling access to and hydrolysis of pPET (Figure 3). This could result in the generation of smaller PET oligomers capable of entering the periplasm, where they may be further degraded by the inner membrane-localized LsrB_m_ variant. To test this hypothesis, both strains (S1 and S3) were cultured in M9 medium supplemented with pPET particles (final concentration, 5 mg mL^−1^) at 37°C for 192 h. During this period, cell growth and the appearance of degradation products were monitored spectrophotometrically by measuring the absorbance at 600 nm (OD_600 nm_) and by HPLC (Figure 4A), respectively.

We observed that the newly engineered strain efficiently degraded pPET at 37°C, reaching 97 ± 1 μM degradation products by 192 h, with concentrations still increasing at the final time point (Figure 4A). The primary degradation products were T, followed by TE (Figure 4A). HPLC chromatograms recorded at 242 nm and at various time points (Figure S5) revealed progressive accumulation of the expected products, whereas additional peaks, particularly at longer retention times, suggested the formation of other PET-derived oligomers. Because these compounds were not fully identified and therefore not quantified, the reported value of 97 ± 1 μM likely underestimates the total extent of PET degradation. These results provide evidence that the engineered *E*. *coli* strain is capable of biodegrading PET powder without the need to introduce any external PETase genes but solely through the recoding of one of its native proteins to function as a PETase.

The engineered strain’s ability to metabolize EG was not validated through direct concentration measurements but rather inferred from the introduction of heterologous EG-metabolizing genes known to sustain cell growth, following approaches commonly reported in the field (Table S1). Indeed, because strain S3 also carries heterologous genes enabling the metabolism of PET degradation products, particularly EG, we were able to assess whether the engineered *E*. *coli* can use PET powder as a carbon source. To test this hypothesis, we monitored cell growth by measuring the absorbance at 600 nm. The final optical density at 600 nm after 192 h was approximately 1.30 ± 0.01, and a maximum growth rate of approximately 0.18 ± 0.07 h^−1^, corresponding to a doubling time of approximately 3.84 ± 1.40 h, was observed (Figure 4A). Notably, these values might be slightly overestimated, as the increase in optical density was likely influenced by the accumulation of partially insoluble PET degradation products that increased turbidity during cultivation. In contrast, strain S1 did not exhibit detectable degradation activity toward pPET particles at any time point or appreciable growth or turbidity, which is consistent with the absence of detectable hydrolysis products. Notably, a small amount of glycerol (0.1 mg mL^−1^) was added during cultivation in all the tests, as glycerol has been reported to promote EG utilization.^43^ However, at this concentration, no appreciable growth was observed in the absence of EG under the tested conditions, as seen for strain S3 in Figure 4A.^25^ It is to highlight also, that although T accumulated in the culture medium (Figure 4A), strain S3 remained metabolically active and capable of hydrolyzing PET powder and sustaining growth, suggesting that this accumulation did not have a detrimental effect on cell viability or growth.

We further investigated pPET degradation in M9 medium supplemented with increasing substrate concentrations while maintaining a constant cell density equivalent to an initial OD_600 nm_ of 0.1. As the pPET load increased from 5.0 to 30 mg mL^−1^, the concentration of degradation products increased exponentially, reaching 157 ± 2 μM after 24 h at 37°C with PET powder (<300 μm, >40% crystallinity) at 30 mg mL^−1^ (Figure 4B).

Finally, to confirm that replacement of OmpA with its recoded variant OmpA_m_ had no detectable impact on cellular fitness, we compared the growth rate of strain S3 in standard Luria-Bertani medium at 37°C (0.633 ± 0.05 h^−1^; raw data in Data S1G) with that of the parental strain S1 (0.627 ± 0.01 h^−1^; measured by Vidal et al.^25^). The resulting selection coefficient (S), calculated as detailed in STAR Methods, indicated less than 1% change in fitness.

## Discussion

This work builds on previous efforts to engineer an *E*. *coli* strain capable of degrading PET nanoparticles without exogenous DNA by introducing artificial serine-histidine-aspartate/glutamate catalytic triads into endogenous proteins and replacing their wild-type counterparts in the genome using CRISPR-Cas9 editing by the recoded variants.^25^ By optimizing our computational design pipeline, which transitioned from the substrate-centered *ligand exploration* to the triad-first *triad exploration* protocol, we systematically increased the number of possible catalytically competent triads while preserving substrate binding. In our earlier work, scaffold selection was guided by strict criteria, including protein size (100–450 amino acids), the presence of signal peptides or transmembrane domains, accurate 3D structural prediction (predicted Local Distance Difference Test [pLDDT] score >70), the identification of binding pockets located ≥6 Å from native catalytic residues with favorable binding energies for model PET substrates, and the ability of engineered Ser-His-Asp triads to remain catalytically competent (interatomic distances <3.5 Å for >35% of simulation time). In contrast, the optimized *triad exploration* protocol presented here builds on our previous method but follows a new approach, systematically identifying geometrically pre-organized catalytic triads across the entire protein surface, not limited to the first identified binding sites. This triad-first strategy integrates stricter geometric and angular constraints, evolutionary tolerance assessments using PSSMs, solvent accessibility checks, preliminary Rosetta-based structural relaxation, and our previously used metrics for PELE and MD triad evaluation, thereby expanding the search space and increasing the likelihood of functional variants. Table 2 summarizes both the methodological and quantitative comparisons between the previous *ligand exploration* and the current *triad exploration* pipelines, highlighting that although the *triad exploration* workflow analyzed a smaller protein subset, it generated a markedly greater number of viable catalytic triads while maintaining a comparable success rate (∼35%).

**Table 2:** Comparison of computational design parameters applied in previous (*ligand exploration*^25^) and current (*triad exploration*) pipelines

Parameter	Ligand Exploration	Triad Exploration (This work)
Protein size (aa)	100–450	All protein length
pLDDT score threshold	>70	>70
Binding pocket distance from native catalytic residues	≥6 Å	≥6 Å
Binding energy thresholds of PELE GE (kcal mol^−1^)	ETETETE < −39.95 ETETE < −32.06 TE < −24.67	Not performed
Catalytic geometry maintenance in MD	>35% of frames	>37% of frames
Angular constraints	–	100 and 180°
Distance constraints (Ser - Lig, Ser - His, His - Asp/Glu) (Å)	Yes: ≤5.0, ≤3.5, ≤3.5	Yes: ≤5.0, ≤3.5, ≤3.5
Residues in triad	Ser–His–Asp	Ser–His–Asp/Glu
Evolutionary tolerance filtering (PSSM)	Not applied	Yes, ≥0
Solvent accessibility check	Yes	Yes
Catalytic triad prerelaxation step	No	Yes
Proteins in the *E*. *coli* genome	4,402	4,402
Proteins selected for analysis	386	43
Possible catalytic triads incorporated (in identified binding sites)	∼450	15,757 (reduced to 397 after fast relax)
Triads after first PELE screening	263	133
Triads selected for MD simulations	68	59
Active variants identified	3	4

The results presented here demonstrate that four of the Ser-His-Asp/Glu catalytic triads designed using the *triad exploration* workflow successfully conferred PETase activity to *E*. *coli* proteins, regardless of the specific conditions under which activity was detected (e.g., temperature) or the type of PET substrate tested (nPET or pPET) (Tables 1 and S4). This corresponds to a significant 33% success rate, comparable to that achieved with the previously used *ligand exploration* protocol.^25^ Importantly, none of the four newly introduced catalytic triads were detected with the first version of GenRewire, underscoring that the new workflow significantly expands the capacity to design artificial PETases by increasing the number of viable candidates. In total, considering both the previous protocol^25^ and the optimized version in the present study, we successfully repurposed seven native proteins from the *E*. *coli* BL21 (DE3) proteome to acquire activity on PET, either nPET, pPET, or soluble PET oligomers (e.g., ETE). Their predicted subcellular localization is illustrated in Figure 3. Although the activities of these variants remain below those of specialized and optimized benchmark PETases such as LCC-ICCG^7^ and HotPETase^20^ (Table 1), particularly under conditions relevant for large-scale PET hydrolysis,^23^ there is considerable room for improvement using additional computational redesign strategies, which were beyond the scope of the present study. Although further efforts will be required to increase the success rate and optimize the newly introduced catalytic centers, it must be emphasized that *de novo* endowing native proteins with the capacity to hydrolyze highly recalcitrant polymers such as PET remains an exceptional challenge in enzyme design, making the achievements reported here significant. Indeed, PET hydrolysis requires highly specific structural features, such as precise catalytic residue placement, substrate-accessible binding pockets, and sufficient flexibility. These are difficult to recreate in an enzyme that has not naturally evolved for polymer degradation. Despite incorporating PSSM-based evolutionary information to minimize destabilizing mutations, engineering the active site and introducing amino acid substitutions could still destabilize the scaffold, and computational tools still struggle to predict how local substitutions affect global structure and enzyme activity.

The results also confirmed that PET powder with particle sizes < 300 μm, which cannot penetrate into the interior of *E*. *coli* because only particles smaller than ∼5.5 nm can pass through outer membrane pores,^44^ can nevertheless be deconstructed *in vivo* by the engineered strain S3, as shown experimentally, without introducing any foreign DNA for this purpose (Figure 4). This strain contains a single endogenous outer membrane protein replaced by a variant (OmpA_m_) and an inner membrane-localized protein replaced by another variant (LsrB_m_), both of which carry three-point amino acid changes that confer PET-hydrolyzing activity. These findings demonstrate that the newly repurposed variant OmpA_m_ has an artificial active site on the cell surface, enabling direct interaction with and depolymerization of the PET powder. This observation aligns with previous studies highlighting the importance of polymer surface accessibility to enzymes and their active sites to promote PET hydrolysis.^45^^,^^46^^,^^47^^,^^48^

Although further biochemical confirmation (e.g., by protease digestion, surface immunofluorescence, or fractionation assays) would be valuable, the ability of the engineered strain to hydrolyze PET powder, which consists of particles far larger than the permeability limit of the outer membrane,^44^ provides functional evidence that the catalytic triad introduced in OmpA_m_ is indeed surface exposed. This direct access to insoluble polymeric substrates implies that the engineered active site is accessible on the external face of the outer membrane, which is consistent with its predicted topology (Figure 2). We have not experimentally validated that the engineered OmpA variant retains all aspects of its original physiological function. However, the growth of engineered bacterial strain in the presence of PET powder suggests that the outer-membrane scaffold remains functionally competent to support cell viability under the tested conditions. In this context, OmpA plays a vital structural role in maintaining outer-membrane stability such that any major impairment of its functionality would likely compromise *E*. *coli* viability.^49^ While this provides a reassuring indication of structural preservation, a full assessment of the native OmpA functions (such as outer-membrane integrity, stress resistance, or peptidoglycan binding) would require dedicated studies beyond the scope of the present work. Notably, in our design, the introduced catalytic triads are located at least 6 Å away from residues involved in structural stabilization or from native catalytic centers (in the case of other *E*. *coli* enzymes repurposed as PET hydrolases), minimizing the risk of perturbing essential structural or functional elements.^25^ In line with this, the *E*. *coli* strain harboring the recoded LsrB variant (LsrB_m_) (strain S1) was shown to exhibit growth fitness comparable to that of the wild-type strain.^25^ Similarly, replacement of OmpA with its recoded variant (OmpA_m_) did not result in any detectable changes in cellular fitness. These findings further suggest that the introduction of PETase-like catalytic functions does not significantly compromise the native roles of the repurposed proteins.

Although not intended to compete with specialized PET-degrading organisms, either native or engineered (see Table S1 and the references cited therein), these findings demonstrate the feasibility of our strategy to confer *E. coli*, a microorganism that does not naturally possess this function, with the ability to degrade large PET particles. In terms of their performance, as shown in Table S1, engineered *Clostridium thermocellum* strains expressing LCC variants through plasmid-based systems or genome *knock-ins* consistently presented the highest reported activities, with ∼12,000–25,000 μM degradation products released from PET films or particles after 240–336 h at 60°C. Similarly, a genome-integrated LCC variant (LCC_I F243I) in *C*. *thermocellum* produced ∼15,000 μM after 240 h at 60°C. Engineered *E*. *coli* MG1655 strains, using the pSEVA238 plasmid to secrete PETases such as chimeric *Is*PETase-*Is*MHETase, FAST-PETase, or PHL7, yielded ∼3,800–4,200 μM from amorphous PET after 504 h at 30°C, whereas *Yarrowia lipolytica* carrying the pAD plasmid to secrete *Is*PETase reached 3,190 μM after 240 h at 28°C. *E*. *coli* PHL628, engineered with a curli display system (pBbE1a-CsgA), produced ∼2,000–3,250 μM from PET powder after 168 h at 30°C–37°C. In comparison, only ∼300 μM of *Ideonella sakaiensis*, which naturally harbors secreted *Is*PETase and *Is*MHETase, was released from PET films after 18 h at 30°C. At the lower end, *Vibrio natriegens* expressing anchored chimeric PETases (*Is*PETase-*Is*MHETase or FastPETase-*Is*MHETase via the pUC19 plasmid) degraded PET powder with very low efficiency, releasing only 3–4 μM after 168 h at 30°C. Our genome-rewired *E*. *coli* BL21 (DE3) sits between these extremes, achieving 157 ± 2 μM from PET powder in just 24 h at 37°C. Notably, unlike all other examples relying on plasmid expression or exogenous constructs, this performance was achieved without the aid of foreign PETase genes, highlighting the potential of genome rewiring for fully endogenous PET biodegradation. Notably, the results reported here were obtained under conditions of an initial OD_600 nm_ of 0.1, a PET powder load of 30 mg mL^−1^, and 37°C, in contrast to other studies that employed resting cell assays with OD_600 nm_ values up to 20, longer incubation times, or higher temperatures (Table S1). In summary, although the biodegradation capacity of the engineered *E*. *coli* strain reported in this study is relatively modest, it is significant given that it was achieved at mesophilic temperature (37°C) on unmodified PET powder and using only rewired endogenous proteins, without the introduction of any exogenous PETase genes.

The engineered strain developed in this study could serve as a novel chassis for future synthetic biology and metabolic engineering strategies aimed at constructing microbial platforms for PET biodegradation and valorization.^50^ To advance this goal, enhancing the performance of artificial PETases designed from *E*. *coli* proteins may be valuable, either as a prerequisite or in parallel. Such improvements could include the computational redesign of their artificially introduced active sites as well as their pockets, substrate tunnels, or protein surfaces, as has been effectively applied to other hydrolases for near-complete PET depolymerization under industrially relevant conditions.^19^ However, this could be challenging because these improvements in PETase activity should occur without affecting the native function of the proteins. Although increasing PETase thermostability is often beneficial, it may not be a priority in *E*. *coli*-based systems because of the temperature limitation of the organism at 37°C. In contrast, introducing the GenRewire strategy into alternative microbial hosts, particularly thermophiles, may allow PET depolymerization at elevated temperatures,^8^ where polymer chains become more flexible and accessible to enzymatic attack by repurposed proteins.^51^

### Limitations of the study

A limitation of this study is that the ability of the engineered strain to metabolize EG was not directly verified through analytical measurements. EG utilization was inferred from the presence and expression of heterologous EG-catabolic genes, which enable its use as a growth-supporting carbon source; however, the actual contribution of EG to cellular metabolism, particularly in the presence of glycerol, could not be mechanistically clarified, as has been reported by others.^42^^,^^43^ In addition, terephthalic acid accumulated in the culture and was not assimilated by the engineered strain, so its impact on overall cellular metabolism has not been verified. Although PET hydrolysis by the engineered strain provides functional evidence of direct access of the engineered catalytic triad in OmpA_m_ to insoluble polymeric substrates, implying that the active site is accessible on the external face of the outer membrane, consistent with its predicted topology, the precise positioning of the active site remains to be experimentally confirmed. These limitations do not affect the conceptual advance demonstrated here but highlight areas where deeper mechanistic validation of both metabolic and structural aspects will be required.

## Resource availability

### Lead contact

Requests for further information and resources should be directed to and will be fulfilled by the lead contact, Manuel Ferrer (mferrer@icp.csic.es).

### Materials availability

Plasmids and bacterial strains generated in this study are available upon request to the lead contact. Identifiers of bacterial strains generated are listed in the key resources table. This study did not generate additional new unique reagents.

### Data and code availability

• The unprocessed HPLC data (Data S2A) and MS files (Data S2B) have been deposited at Zenodo under the identifier https://zenodo.org/records/17578239. The directory contains separate folders for each type of data, and README files. The Molecular Dynamics Simulations data have been deposited at Zenodo under the identifier https://doi.org/10.5281/zenodo.17052114. To use the archive, download the file, and extract its contents to a local directory using appropriate software. The directory contains separate folders for each type of simulation, along with input, output and README files.
• This article does not report original code.
• All other data reported in this manuscript will be shared by the lead contact upon request.

## Acknowledgments

The authors (M. Ferrer and V.G.) thank the European Union’s Horizon 2020 Research and Innovation Programme for grant 101000327-FuturEnzyme, as well as the 10.13039/100018693Horizon Europe for grant 101060625-Nymphe (M. Ferrer). They also acknowledge funding from the Ministerio de Ciencia, Innovación y Universidades, 10.13039/501100011033Agencia Estatal de Investigación (AEI) (MICIU/AEI/10.13039/501100011033), 10.13039/501100002924FEDER, EU, and the European Union NextGenerationEU/PRTR, supporting projects PID2020-112758RB-I00 (M. Ferrer), PDC2021-121534-I00 (M. Ferrer), TED2021-130544B-I00 (M. Ferrer), PID2023-153370OB-100 (M. Ferrer), PID2019-106370RB-I00 (V.G.), and PID2022-136367OB-C31 (F.J.P.). Funding from the “César Nombela” Research Talent Attraction Program, grant 2024-T1/ECO-31227 by the Consejería de Educación, Ciencia y Universidades de la Comunidad de Madrid (R.B.) and European Union’s HORIZON-MSCA-2022-PF-01 program grant 101104264-BIO DEGRADE (J.G.-D.) are also gratefully acknowledged.

## Author contributions

Conceptualization was led by V.G. and M. Ferrer. Methodology was developed by P.V., L.F.-L., D.A., J.G.-D., S.R., M.L., M.M.-S., A.R.-M., M. Floor, R.B., and J.L.G.-A. Investigation was carried out by P.V., L.F.-L., and D.A. Visualization efforts were contributed by J.G.-D., S.R., L.F.-L., M.M.-S., and A.R.-M. Supervision was provided by V.G., M. Ferrer, L.F.-L., R.B., and F.J.P. Writing – original draft was undertaken by V.G., L.F.-L., M. Ferrer, and J.G.-D., while writing – review and editing were handled by V.G., M. Ferrer, L.F.-L., and P.V.

## Declaration of interests

The authors declare no competing interests.

## Declaration of generative AI and AI-assisted technologies in the writing process

During the preparation of this work, ChatGPT or other generative AI tools were not used. The authors take full responsibility for the content of the manuscript, which, once completed, was reviewed and edited for language by a professional editing service.

## STAR★Methods

### Key resources table

**Table:** 

REAGENT or RESOURCE	SOURCE	IDENTIFIER
**Bacterial and virus strains**		

*E. coli* BL21 (DE3)	Agilent Technologies, Inc.	200131
*E. coli* BL21 (DE3) *ΔlsrB::lsrB*_*m*_	GenScript Biotech (Netherlands) B.V.	U062JMDUG0
*E. coli* BL21 (DE3) Δ*lsrB* Δ*ompA*::*lsrB*_m_*ompA*_m_	GenScript Biotech (Netherlands) B.V.	U023X115G0

**Chemicals, peptides, and recombinant proteins**		

Luria Bertani (LB) medium	ThermoFisher Scientific	12780029
LB agar medium	ThermoFisher Scientific	22700041
M9 minimal medium	Merck Life Science S.L.U.	M6030
Ampicillin	ThermoFisher Scientific	BP1760
Isopropyl β-D-1-thiogalactopyranoside	ThermoFisher Scientific	302790250
Glycerol, ≥99.5% (GC)	Merck Life Science S.L.U.	49767
D-(+)-Glucose, ≥99.5%	Merck Life Science S.L.U.	G7021
NaH_2_PO_4_, 99-100,5%	Panreac Química S.L.U.	141677.1211
Na_2_HPO_4_, 99-102 %	Panreac Química S.L.U.	131678.1211
NaCl, ACS reagent, ≥99.0%	Merck Life Science S.L.U.	106404
4-(2-Hydroxyethyl)-1-piperazineethanesulfonic acid, 99%	ThermoFisher Scientific	BP310-5
Imidazole, ≥99.5% (GC)	Merck Life Science S.L.U.	56748
Bis(2-hydroxyethyl) terephthalate	Merck Life Science S.L.U.	465151
Solvent pre-treated amorphous PET	Goodfellow Cambridge	ES301445
PET powder (<300 μm, >40% crystallinity)	Goodfellow Cambridge	ES30-PD-000132
1,1,1,3,3,3-Hexafluor-2-propanole	Merck Life Science S.L.U.	105228
Formic acid, 85% w/w	Scharlab S.L.	AC1080
Acetonitrile, min. 99.9 %	Scharlab S.L.	AC0371
Dimethyl sulfoxide, 99,9%	Scharlab S.L.	SU01521000
Trifluoroacetic acid, purum, ≥98.0% (T)	Fluka	91700
Lysonase Bioprocessing reagent	Merck Life Science S.L.U.	71230
HIS-Select Nickel Affinity Gel	Merck Life Science S.L.U.	P6611
Amicon® Ultra-15 centrifugal filter unit, regenerated cellulose membrane, MWCO 10 kDa	Merck Life Science S.L.U.	UFC9010
Nylon syringe filter, 0.45 μm pore size, 13 mm diameter	Membrane Solutions	SFNY013045N
Syringe filters with acrylic housing sterile, 0.2 μm pore size, 25 mm diameter	VWR International Inc.	28145-501
SeeBand protein staining solution	Gene Bio-Application Ltd.	SB010
Bio-Rad protein assay dye reagent concentrate	Bio-Rad Laboratories	500-006
Superdex 75 10/300 GL and 5/150 GL	Merck Life Science S.L.U.	GE17-5174-01
Zorbax Eclipse Plus C-18 (4.6 x 100 mm, 3.5 μm)	Agilent Technologies, Inc.	959961-902
Zorbax Eclipse XDB C-18 column (9.4 × 250 mm, 5 μm)	Agilent Technologies, Inc.	990967-202
Sunfire C18 RP precolumn (2.1 mm × 5 mm)	Waters Corporation	186002539
Atlantis T3 C18 RP column (2.1 mm × 100 mm)	Waters Corporation	186003718

**Recombinant DNA**		

pET-45b(+) plasmids	GenScript Biotech (Netherlands) B.V.	U884RPCAG0 U9448751G0

**Software and algorithms**		

SignalP 5.0	DTU Health Tech	https://services.healthtech.dtu.dk/services/SignalP-5.0/
DeepTMHMM	Technical University of Denmark	https://dtu.biolib.com/DeepTMHMM
AlphaFold Protein Structure database	Google DeepMind	https://alphafold.ebi.ac.uk/
AlphaFold 2.0	Google DeepMind	https://alphafold.ebi.ac.uk/
Protein Preparation Wizard	Schrödinger	https://www.schrodinger.com/life-science/learn/white-papers/protein-preparation-wizard/
PELE Software (version 1.8.0), PELE Platform 1.6.3, AdaptivePELE version 1.7.1	Electronic and Atomic Protein Modeling (EAPM) group, BSC	https://pele.bsc.es/pele.wt https://nostrumbiodiscovery.github.io/pele_platform/# https://nostrumbiodiscovery.github.io/pele_docs/
Rosetta modeling software suite (release 337)	Rosetta Commons	https://rosettacommons.org/software/
GROMACS 2018	GROMACS	https://manual.gromacs.org/2018/index.htmL
Antechamber Python Parser interfacE (ACPYPE)	https://alanwilter.github.io/acpype/	https://www.bio2byte.be/acpype/
MDtraj	Stanford University	https://www.mdtraj.org/1.9.8.dev0/index.htmL
PyMOL	Schrödinger	https://www.pymol.org/
Matplotlib	Matplotlib	https://matplotlib.org/
Seaborn	Seaborn	https://seaborn.pydata.org/
WSxM free software package	WSxM	http://wsxm.eu/
ImageJ free software package	National Institutes of Health	https://imagej.net/ij/download.htmL
Varian Star LC workstation 6.41	Agilent Technologies, Inc.	https://www.agilent.com/library/usermanuals/public/914760.pdf
MassLynx V4.1	Waters Corporation	https://www.waters.com/webassets/cms/support/docs/71500113203ra.pdf
R programming environment	The R Foundation	https://www.r-project.org/
BioTek Gen5 2.0 software	Agilent Technologies, Inc.	https://www.agilent.com/en/product/microplate-instrumentation/microplate-instrumentation-control-analysis-software/imager-reader-control-analysis-software/biotek-gen5-software-for-detection-1623227

**Others**		

Proteome database	UniProt	https://www.uniprot.org/
Proteome database	UniParc	https://www.uniprot.org/uniparc?query=∗
Proteome database	STEP	http://httpd.apache.org/
Proteome database	EchoLOCATION	http://www.ecoli-york.org/
Bioinformatics Resource	ExPASy	https://web.expasy.org/compute_pi/
Burette	Afora S.A.	A-6055
Screw tube 15 mL, 120 x 17 mm	Sarstedt AG & Co.	62.554.502
Screw tube 50 mL, 114 x 28 mm	Sarstedt AG & Co.	62.547.254
2-mL Safe-lock polypropylene tubes	Eppendorf SE	0030120094
96-Well U-bottom microplate	Greiner Bio-One	650161
Vial of 2 mL clear 9-425 screw top	Membrane Solutions	LBSV022C
0.25 mL micro-insert, 31 x 6 mm, conical, clear glass, 15 mm top	Lab Logistic Group GmbH	7401744
3 Parts syringe without needle	Ico Plus 3	N14360
Vial caps, pre-slit blue PTFE/White silicone septa+ blue screw cap with hole, for 2 mL	Membrane Solutions	LBSV222CSS
Pur-A-Lyzer™ Maxi 1200 dialysis kit	Merck Life Science S.L.U.	PURX60100
Rotavapor R-210	Buchi Ibérica S.L.U.	CH9230
Sonicator 3000	Misonix, Inc.	M98820
Fast protein liquid chromatography (FPLC)	Amersham Bioscience	LCC-500CI
Mini PROTEAN electrophoresis system	Bio-Rad Laboratories	041BR
MaxQ 6000 refrigerated incubator shaker	Thermo Fisher Scientific	T-MAXQ6
Centrifuge 5810 R	Eppendorf SE	581112714
Orbital incubator SI500	Stuart Scientific Co. Ltd	R000102971
BioPhotometer	Eppendorf SE	6131 25728
GenePulser Xcell™	Bio-Rad Laboratories	1652667
Quaternary pump Model 1100	Agilent Technologies, Inc.	https://www.agilent.com/en/support/liquid-chromatography/a03681
Autosampler Model L-2200	Hitachi, Ltd.	890-0202
Photodiode array detector Varian ProStar	Varian Inc.	EL05059010

### Experimental model and study participant details

#### Bacterial strains

*E. coli* strains used in this study are listed in the key resources table. Experiments were performed using *E. coli* BL21 (DE3) and CRISPR–Cas9–engineered derivatives carrying the *lsrB*_*m*_ and *ompA*_*m*_ genes. *E. coli* BL21 (DE3) was supplied by Agilent Technologies, Inc. (ref. 200131) and was used as heterologous expression hosts. Strains *E. coli* BL21 (DE3) *Δlsr*B::*lsrB*_m_ and *E. coli* BL21 (DE3) *Δlsr*B *Δomp*A::*lsrB*_m_ *omp*A_m_ were generated by genome editing by GenScript Biotech (Netherlands) B.V. (ref. U062JMDUG0 and U023X115G0, respectively) and used for functional validation. All strains were cultured under standard laboratory conditions using Luria Bertani (LB), M9 minimal medium or defined media as specified in the Methods. Cultures were grown at 37°C with appropriate antibiotic selection where required. No animals, human participants, plants, cell lines, or primary cell cultures were used in this study.

### Method details

#### Structure generation, binding site scanning, molecular dynamics simulations

##### *E*. *coli* secreted protein selection and structure prediction

Using literature sources and proteome databases, we manually searched for proteins reported to be secreted in *E*. *coli*.^34^^,^^35^^,^^36^ We retrieve the 3D crystal structures or models of the protein sequences from the RCSB PDB (RSCB.org) or the AlphaFold Protein Structure^52^^,^^53^ delivery of experimentally-determined PDB structures alongside one million computed structure models of proteins from artificial intelligence/machine learning database,^54^^,^^55^ respectively, if available, or running AlphaFold 2.0.^56^ AlphaFold models were filtered to remove those with average per-residue model confidence score (pLDDT) scores below 70, but all the 43 models were retained. All remaining protein models were prepared with the Protein Preparation Wizard from Schrödinger^57^ to fix protonation states at pH 7.5 and other common problems (missing side chains, atomic clashes, etc.).

##### Catalytic Ser–His–Asp/Glu triad design

For each protein sequence a PSI-BLAST (Position-Specific Iterated BLAST) search was conducted to identify homologous sequences and generate evolutionary information. The searches were performed against the TrEMBL database. PSI-BLAST was iterated for five rounds using an E-value threshold of 0.001, obtaining a maximum of 5000 sequences. The resulting multiple sequence alignments (MSA) were used to construct PSSMs, which encode the probability of observing each amino acid at every position of the sequence, reflecting the evolutionary conservation and variability across related sequences. Subsequently, these PSSMs were used as input features in the catalytic triad design in the next step to evaluate the mutational probability when introducing catalytic triad residues. The modified triad design protocol systematically scanned all surface-exposed residues of the target protein for potential serine and histidine positions. For each such position, all available rotamers were evaluated at 20° increments. Rotamer pairs exhibiting catalytic distances between serine and histidine (distance Ser O - His NE2/ND1 ≤ 4.0 Å) and an angular constraint between 100° and 180° (Ser OG - Ser HG - His NE2/ND1) were accepted. Subsequently, neighboring positions to these histidine residues were explored for compatible aspartic or glutamic acid residues, with catalytic distances between histidine and aspartic or glutamic acid (distance His HD1/HE2- Asp OD1/OD2 or Glu OE1/OE2 ≤ 4.0 Å) and angular constraint between 100 and 180° (His NE2/ND1 - His HD1/HE2 - Asp OD1/OD2 or Glu OE1/OE2). The angular constraints were chosen after calculating the angular constraints of all serine hydrolases in the PDB. The previously calculated PSSM conservation scores were then used to assess the mutational tolerance of each candidate residue position; only positions with a tolerance score greater than zero were considered. Finally, from all geometrically compatible rotamer combinations, the one with the lowest Rosetta score^58^ was selected for each unique triad identified. All selected triads underwent structural relaxation using Rosetta’s *FastRelax* protocol from Rosetta Modeling Software to remove potential steric clashes introduced by the newly mutated residues, and evaluate catalytic distances and angles of the introduced triad. This relaxation was performed only on the catalytic residues and neighbors within a 12 Å distance, with the protein having positional constraints on backbone and side-chain heavy atoms, employing a constraint ramp from full to zero during the initial phase. For each model, five relaxation cycles with 30 independent trajectories were generated. This number was selected, following a small-scale test, as it provided an optimal balance between having a sufficiently large sample size to determine whether the catalytic triad was geometrically organized, and managing the computational cost associated with evaluating a high number of possible triads. We calculated the distances and the angle formed by the residues within each identified catalytic triad in all the relaxed independent trajectories. Only models exhibiting triad residues positioned at catalytically relevant distances (distance Ser - His ≤ 4 Å, distance His - Asp/Glu equal or less than 4 Å) in more of the 50% of the trajectories for each model were retained for subsequent analysis. The pose with the best Rosetta score was retained, as determined in a retrospective small test with previous PluriZymes.^25^^,^^27^^,^^28^^,^^29^^,^^30^ The designed catalytic triads were also filtered based on their coplanarity and collinearity, with acceptable ranges for these geometric features defined by analysis of wild-type PETases and serine hydrolases with solved structures in the PDB.

##### Docking glide protocol

To perform ligand docking, we used Glide^59^ from the Schrödinger suite [Schrödinger Release 2021-4: Glide; Schrödinger, LLC: New York,NY, 2021]. A PET trimer molecule (ETETETE) was docked into the newly engineered active sites of each protein, selecting the center of the docking grid box to be the oxygen atom from the hydroxyl group of the catalytic Serine. Then we modified the inner box to cover the volume of the entire ligand, while the dimensions of the outer box were generated automatically. The best scoring Glide pose that fulfil catalytic distances (distance Ser O - His NE2/ND1 ≤ 3.5 Å, distance His HD1/HE2- Asp OD1/OD2 or Glu OE1/OE2 ≤ 3.5 Å, distance Ser O - Lig C <5A) was considered to follow the protocol.

##### Local refinement

To evaluate the binding and potential catalytic activity of the designed variants, we performed substrate-induced fit simulations using the Protein Energy Landscape Exploration (PELE) software (version and citation if available).^60^^,^^61^^,^^62^ ETETETE was employed as a model substrate to mimic the polymer binding. PELE is an unconstrained ligand exploration technique based on Monte Carlo sampling. The algorithm initiates by performing a substrate perturbation (translation and rotation), and then it predicts the protein side-chain conformations within 6 angstroms of the substrate using an experimental rotamer library. Then, it performs a final minimization to generate a new pose, which is accepted or rejected according to the Metropolis criterion. For each designed protein variant incorporating the catalytic triad, we conducted induced fit simulations with the PET trimer substrate. The simulations were performed in two consecutive rounds. The first round consisted of 100 steps, 5 iterations, running in 56 independent trajectories. Following this initial exploration, the resulting poses were evaluated based on a series of metrics describing the hydrolysis process. We used the following metrics: distance of the C atoms of the carbonyl group/s of the substrate to the O atom of the catalytic serine (Ser - Lig) below 5 Å, distance of the O atom of the catalytic serine to the deprotonated N atom of the catalytic histidine (Ser - His) below 3.5 Å, and distance of the protonated N atom of the catalytic histidine to the closest side chain O atom of the catalytic aspartic glutamic acid (His - Asp/Glu) below 3.5 Å. Poses exhibiting favorable binding energies and meeting the defined metric requirements were selected as starting points for the second, more computationally intensive simulation round. This second round involved 100 steps, 10 iterations, and utilized 112 CPUs to achieve higher sampling of the conformational space. Designed catalytic triads were filtered based on the results of their PELE scores before further assessment with molecular dynamics. Following our previously established methodology,^25^ we defined the catalytic binding free energy as the expectation value of the binding energy for catalytic poses, calculated based on a Boltzmann distribution, and used it as the filtering score. First, probabilities were assigned to each pose in the PELE simulation according to a Boltzmann distribution:

$$P_{i}=\frac{e^{-E_{i}/KT}}{Q}$$

Here, Pi is the probability of the ith pose, Ei is its total energy, KT is the energy partition constant, and Q is the partition function for a simulation of N poses, defined as:

$$Q=\sum_{i}^{N}e^{-E_{i}/KT}$$

Finally, the above probabilities are used to integrate the individual binding energy values Eib of the catalytic poses of the simulation:

$$<E^{b}>=\sum_{i}^{N}P_{i}E_{i}^{b}$$

##### Molecular dynamics

We performed MD simulations with GROMACS 2018,^63^^,^^64^ following our previously established methodology.^25^ Briefly: the systems were built using the AMBER99SB∗_ILDN force field^65^ for proteins and TIP3P water,^66^ while ligands (ETETETE) were parameterized with ACPYPE using GAFF2 and the AM1-BCC scheme.^67^^,^^68^^,^^69^ Systems were neutralized with Na^+^/Cl^-^ ions and solvated in a TIP3P octahedral box. Simulations ran at 298.15 K and 1 bar in the NPT ensemble, with periodic boundary conditions and Particle Mesh Ewald electrostatics. Hydrogen mass repartitioning and LINCS constraints enabled a 4 fs timestep.^70^^,^^71^ Equilibration was performed in NVT and NPT ensembles with gradually decreasing restraints, followed by two 200 ns replicas per system. Catalytic triad distances were analyzed using MDTraj, selecting candidates maintaining a catalytic geometry for more than 37% of the simulation time. Structural representations were made with PyMOL, and graphs with Matplotlib and Seaborn.

#### Production of wild type and mutant proteins

Once identified, the amino acid sequences encoding the wild-type and mutant proteins (see accession numbers and sequences in Table S3) were used as templates for gene synthesis. The sequences were synthesized by GenScript Biotech (GenScript Biotech, EG Rijswijk, The Netherlands) and codon-optimized to maximize expression in *Escherichia coli*. In brief, before gene synthesis, the sequence was analyzed for the presence of a signal peptide using the SignalP-5.0 tool.^72^ The genes, excluding the signal peptide when present, were flanked by BamHI and HindIII (stop codon) restriction sites and inserted into a pET-45b(+) expression vector with an ampicillin selection marker (GenScript Biotech, Rijswijk, The Netherlands). This plasmid, which was introduced into *E. coli* BL21(DE3), supports the expression of N-terminal His6-fusion proteins, with the final amino acid sequence of the synthetic protein being MAHHHHHHVGTGSNDDDDKSPDPM-X (where X corresponds to the original sequence of the target enzyme without the signal peptide). After synthesis, the soluble N-terminal His6-tagged protein was produced and purified at >98% purity, as determined by SDS-PAGE analysis (Figures S1 and S2) using a Mini PROTEAN electrophoresis system (Bio-Rad, Madrid, Spain) at 4°C after binding to a HIS-Select Nickel Affinity Gel (Merck Life Science S.L.U., Madrid, Spain), and stored at -20°C until use at a concentration of 1 mg mL^-1^, as previously described.^25^

#### *In vitro* PET hydrolysis assays

*In vitro* PET hydrolysis assays using ETE, nPET and pPET were performed following the reaction conditions and procedures previously described.^25^ Briefly, standardized nPET substrates were prepared from amorphous PET (GoodFellow Cambridge, Huntingdon, UK; ref. ES301445) as described, and PET powder was obtained from the same supplier (ref. ES30PD000132), while ETE was purchased from Merck Life Science S.L.U. (ref. 465151). Enzymatic reactions were conducted under controlled conditions, including defined buffer composition, temperature, agitation speed, and reaction time, along with appropriate negative controls. Degradation products were quantified by HPLC.^25^ All experiments were performed in biological triplicates (*n = 3*).

#### CRISPR-Cas9 genome editing

The strain *E*. *coli* BL21 (DE3) Δ*lsrB* Δ*ompA*::*lsrB*_m_ *ompA*_m_ / pET-45b(+)[*aldA*, *fucO*] (strain S3) was constructed using *E. coli* BL21 (DE3) Δ*lsrB*::*lsrB*_m_ / pET-45b(+)[*aldA*, *fucO*] (strain S1),^25^ as the starting strain. The strain was engineered and provided by GenScript Biotech (EG Rijswijk, The Netherlands) following the procedures previously described.^25^ The CRISPR-Cas9 edited *E. coli* BL21 (DE3) strain has been provided by GenScript Biotech (EG Rijswijk, The Netherlands) and is available upon request.

#### Preparation and cultivation of engineered *E*. *coli* strains and *in vivo* PET hydrolysis assays

The protocol used for *in vivo* PET hydrolysis using pPET was adapted from the procedure previously developed for nPET degradation assays,^25^ with minor modifications to accommodate the physicochemical properties of pPET. In brief, selected *E*. *coli* BL21 (DE3) clones were grown at 37°C on LB agar plates (ThermoFisher Scientific, MA, USA; ref. 22700041) supplemented with 0.1 mg mL^-1^ ampicillin (ThermoFisher Scientific; ref. BP1760). A single colony was picked to inoculate 10 mL of LB broth containing the same antibiotic in a 50-mL Falcon tube and incubated overnight at 37°C with shaking at 100 rpm. Cells were harvested by centrifugation at 4,000 rpm for 15 min at room temperature, washed twice with sterile M9 minimal medium, and resuspended in 10× concentrated M9 medium to an OD_600 nm_ of approximately 1.0. All experiments were performed in biological triplicates (*n = 3*).

For growth tests (biological triplicates), in parallel, 50 mg of PET powder (pPET) were weighed into a sterile 50-mL Falcon tube. Then, 1 mL of the resuspended bacterial suspension was added, followed by 9 mL of sterile water, 0.1 mg mL^-1^ ampicillin, 1 mM IPTG (to induce *fucO* and *aldA* expression), and 100 μL of a 10 mg mL^-1^ glycerol stock solution. Final assay conditions were: 5 mg mL^-1^ pPET, 0.1 mg mL^-1^ glycerol, and an initial OD_600 nm_ of 0.1. Cultures were incubated at 37°C at 200 rpm. At selected time points, 700 μL aliquots were withdrawn. OD_600 nm_ measurements were recorded using up to 20 consecutive readings per sample to ensure stabilization and minimize interference from PET particles. For quantification of PET hydrolysis products, samples were concentrated using a Concentrator 5301 (Eppendorf®) and resuspended in 200 μL of DMSO.

To evaluate the effect of different pPET concentrations, 2.5, 5, 10, and 15 mg of pPET were weighed into sterile 2-mL Eppendorf® tubes. Then, 500 μL of the resuspended bacterial suspension at initial OD_600 nm_ of 0.1 was added, followed by 0.1 mg mL^-1^ ampicillin, 1 mM IPTG and 100 μL of a 10 mg mL^-1^ glycerol stock solution. Final assay conditions were: 5, 10, 20 or 30 mg mL^-1^ pPET and 0.1 mg mL^-1^ glycerol. Cultures (biological triplicates) were incubated at 37°C and 1200 rpm in ThermoMixer C (Eppendorf®). At 24 h, samples were concentrated using a Concentrator 5301 (Eppendorf®) and resuspended in 200 μL of DMSO.

#### Fitness analysis

The fitness analysis of *E. coli* strain S3 was performed as described by Vidal et al.,^25^ with minor modifications. Briefly, the strain was maintained at –80°C in LB broth supplemented with 20% (v/v) glycerol. For growth assays, a single colony was inoculated into 10 mL of LB medium containing ampicillin (0.1 mg mL^-1^) and incubated overnight at 37°C and 1000 rpm in an orbital shaker (Thermo Scientific MaxQ 6000, Thermo Fisher Scientific, USA). Cells were harvested by centrifugation at 5000 g for 15 min (Eppendorf 5810 R, Eppendorf®, Germany), washed three times with M9 medium, and resuspended to an optical density at 600 nm (OD_600 nm_) of 1.0 using a BioPhotometer (Eppendorf AG, Hamburg, Germany). For microplate growth assays, 180 μL of LB medium supplemented with 1 mM IPTG and 0.1 mg mL^-1^ ampicillin and 20 μL of the OD_600 nm_ = 1.0 cell suspension were added to each well of a sterile 96-well U-bottom microplate (Greiner Bio-One, Austria), resulting in a final OD_600 nm_ of 0.1 (200 μL total volume per well). Cultures (17 replicates) were incubated at 37°C with continuous shaking at 800 rpm, and OD_600 nm_ readings were recorded every 10 min for 24 h using a BioTek Synergy H1 Multimode Reader (Agilent, USA). Raw optical density data were processed using Gen5 2.00 software (BioTek Instruments, USA), and specific growth rates were calculated from the linear interval of the exponential growth phase. The selection coefficient (S) was determined from the growth rate data as described by Vidal et al.^25^

#### UHPLC-MS analysis of PET hydrolysis products

In all cases, HPLC with well-characterized standards and UHPLC-MS, were used for product detection and quantification, following previously published procedures.^25^ In the particular case of dimeric hydrolysis products resulting from PET degradation, UHPLC-MS was employed. Reaction samples were diluted 10 times with DMSO and analyzed by UHPLC-MS via a Waters Acquity arc Separations Module coupled to a Waters 2998 Photodiode Array Detector and coupled to a single quadrupole Waters SQD2 mass spectrometer (Waters Cromatografía, SA; Barcelona, Spain). The compounds were analyzed by reverse phase chromatography using a 2.1 mm × 5 mm Waters Sunfire C18 RP precolumn and a 2.1 mm × 50 mm Waters Sunfire C18 RP column operating at 0.7 mL min^-1^. The injection volume of the sample was 3 μL. The compounds were eluted using a 10 min gradient from 15 to 95% solvents A-B (solvent A: acetonitrile, solvent B: water), which included a 5% fixed percentage of solvent C (solvent C: 2% formic acid in water), with the concentration of formic acid in all the gradients being 0.1%. After separation, the sample was analyzed by optical detection from 190-700 nm with a resolution of 1.2 nm by a Waters 2998 PDA Detector and by a mass analyzer (Waters SQD2Detector) with an electrospray (ES) interface in positive and negative modes with a capillary voltage of 3.5 kV and cone voltages of 20 V and 40 V, with a probe temperature of 500°C and a source temperature of 120°C. The compounds, particularly TET and TETE, were detected via full-scan MS from 85 to 3072 m/z.

### Quantification and statistical analysis

Standard deviations were calculated via the STDEV. S function in Excel 2024. Statistical analyses were performed using the GraphPad Prism 10.0. All experiments were performed with three replicates, and the error bars in the figure legends and tables represent the means ± standard deviations.

## Supplemental information

Document S1. Figures S1–S5 and Tables S1, S4, and S5

Table S2. *E. coli* proteins selected as candidates for catalytic triad engineering Proteins from EchoLOCATION and STEP databases and proteomic studies, including outer membrane β-barrel, secreted, cell-surface, periplasmic, and cytoplasmic proteins, selected to validate the *Triad Exploration* protocol. Of the 43 secreted proteins analyzed in this study, 19 had been previously evaluated with the earlier version of GenRewire and are indicated with an asterisk in the table. UniParc and UniProt accession numbers, gene names, alternative gene names, EchoLOCATION identifier (EchoBASE id), subcellular localization (EchoLOCATION), Evidence level for subcellular localization, predicted molecular weight, number of transmembrane helices (HMMTOP/TMHMM), and signal peptide cleavage probability, are shown in the table.

Table S3. Protein sequences and associated expression system information The table includes accession numbers (with database links), gene names, annotations, and predicted subcellular localizations, based on the EchoLOCATION, STEP and UniProt databases. It shows original and synthetic protein (native and engineered variants) sequences (signal peptides in blue, SignalP-5.0), pET-45(+) plasmid constructs synthesized by GenScript Biotech (EG Rijswijk, The Netherlands; ref. U884RPCAG0, and U9448751G0), antibiotic resistance (ampicillin), inducer (IPTG), molecular weights (calculated with ExPASy; https://web.expasy.org/compute_pi/), and the catalytic triad residues with engineered amino acid substitutions.

Data S1. Raw data for *in vitro* and *in vivo* tests (A–G) Datasets include concentrations of degradation products and the corresponding specific activity values obtained after incubation of ETE, nPET, and pPET with purified engineered enzyme variants (*in vitro* tests). They also include growth data (measured spectrophotometrically at 600 nm) of engineered strain S1 with pPET and Luria-Bertani medium, and degradation product concentrations from *in vivo* cultivations of engineered strains with pPET, under various temperature and incubation conditions (specified for each test). Quantification was performed by HPLC using calibration curves (original chromatograms in Data S2A). Data are provided in triplicates, with both raw and processed values (including applied dilutions or concentration factors). (A–C) Datasets for *in vitro* degradation tests using ETE (A), nPET (B) and pPET (C), including concentrations of degradation products and the corresponding specific activity values. Related to Figure S3, Tables 1 and S4. (D–E) Growth (OD_600 nm_) (D) and concentration of degradation products (E) datasets from *in vivo* cultivations of strains S1 and S3 on pPET as carbon source. Related to Figure 4A. (F) Concentration of degradation products datasets from *in vivo* cultivations of strains S1 and S3 on different concentrations of pPET. Related to Figure 4B. (G) Growth (OD_600 nm_) datasets from *in vivo* cultivation of strain on Luria-Bertani medium.

Data S2. Raw HPLC and UHPLC-MS data (A) Unprocessed HPLC data files supporting Figures 4, S3, S5, Tables 1 and S4. Files are provided in.txt format corresponding to the data extracted with Varian ProStar software (Varian Inc., Palo Alto, California, USA). Each file contains absorbance values (in arbitrary units) recorded over time under the analytical conditions described in STAR Methods. (B) Raw UHPLC-MS files supporting Figure S4. The chromatograms and mass spectrometry data can be analyzed with the software MassLynx V4.1 (Waters) to obtain the mass spectra of the oligomers TET and TETE. Analytical conditions described in STAR Methods. (A and B) The unprocessed HPLC and UHPLC-MS files have been deposited at Zenodo under the identifier https://zenodo.org/records/17578239.
