# Supplementary material for: Engineering Escherichia coli for polyethylene terephthalate powder biodegradation via recoding of an outer membrane protein
Source: iScience. 2026 Jan 2;29(2):114621. doi: 10.1016/j.isci.2025.114621 (PMC12860643; doi:10.1016/j.isci.2025.114621)
Supplement: Document S1. Figures S1–S5 and Tables S1, S4, and S5 [file mmc1.pdf]

iScience, Volume *29*

Supplemental information

Engineering *Escherichia coli* for polyethylene terephthalate powder biodegradation via recoding of an outer membrane protein

Joan Giménez-Dejoz, Paula Vidal, Sonia Romero, David Almendral, Miguel Luengo, Mireia Martinez-Sugrañes, Jose L. Gonzalez-Alfonso, Ana Robles-Martín, Francisco J. Plou, Rafael Bargiela, Martin Floor, Manuel Ferrer, Víctor Guallar, and Laura Fernandez-Lopez

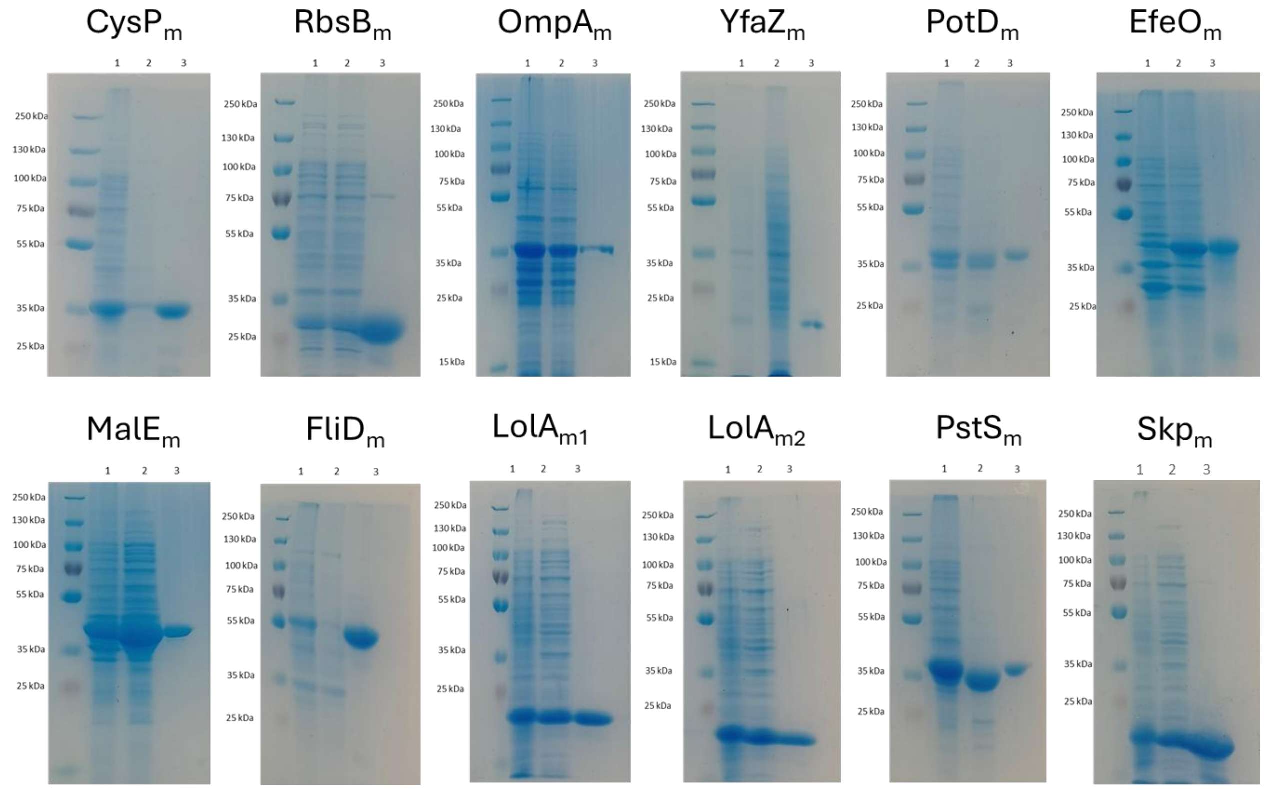

Figure S1. SDS-PAGE analysis of target *E. coli* protein variants (denoted with the subscript m) using a 12% Tris-glycine SDS-PAGE gel

Each gel includes a molecular weight marker (leftmost lane), the total protein fraction (lane 1), the soluble protein fraction (lane 2), and 10 µg of purified protein (lane 3). As shown, expression of the N-terminal hexahistidine-tagged synthetic proteins confirmed that all 12 variants were produced in soluble form. Yield of protein production in 50 mL *E. coli* cultures: CysP$_m$, 2.86 mg; RbsB$_m$, 0.09 mg; OmpA$_m$, 0.46 mg, YfaZ$_m$, 0.16 mg, PotD$_m$, 6.91 mg; EfeO$_m$, 4.55 mg; MalE$_m$, 3.74 mg, FliD$_m$, 2.48 mg; LolA$_{m1}$, 4.08 mg; LolA$_{m2}$, 5.12 mg; PstS$_m$, 1.80 mg; Skp$_m$, 0.06 mg.

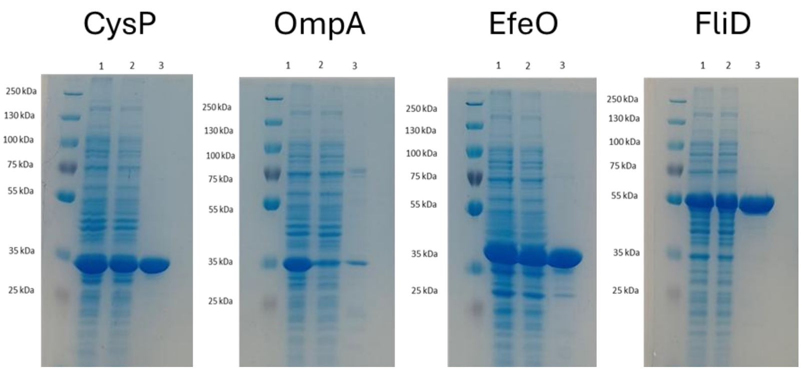

Figure S2. **SDS-PAGE analysis of target *E. coli* wild type proteins using a 12% Tris-glycine SDS-PAGE gel**

Each gel includes a molecular weight marker (leftmost lane), the total protein fraction (lane 1), the soluble protein fraction (lane 2), and 10 µg of purified protein (lane 3). As shown, expression of the N-terminal hexahistidine-tagged synthetic proteins confirmed that all four wild-type proteins, from which the four PETase-active mutants were derived, were produced in soluble form.

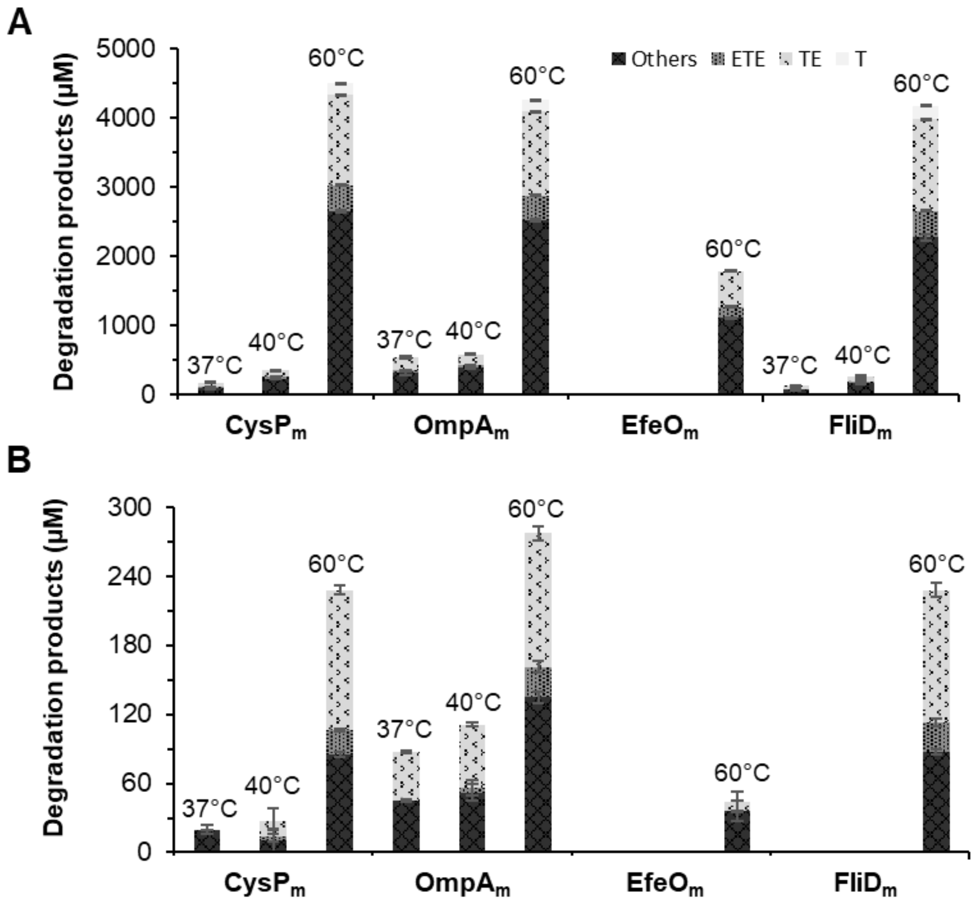

Figure S3. ***In vitro* degradation of nPET and pPET at 24 h**
(A) Concentration of degradation products from nPET by *E. coli* engineered proteins.
(B) Concentration of degradation products from pPET by *E. coli* engineered proteins. Reactions were performed at 37, 40 and 60°C and pH 7.0 (for methods details see legend Table 1 and STAR★METHODS).
(A,B) Degradation products were quantified by HPLC; raw data are available in Data S1. Values (Data S1B for panel A and Data S1C for panel B) represent the means of three biological replicates ($n = 3$), with error bars showing standard deviations (SDs), calculated using the STDEV.S function in Excel 2024. The figure was generated using Excel 2024.

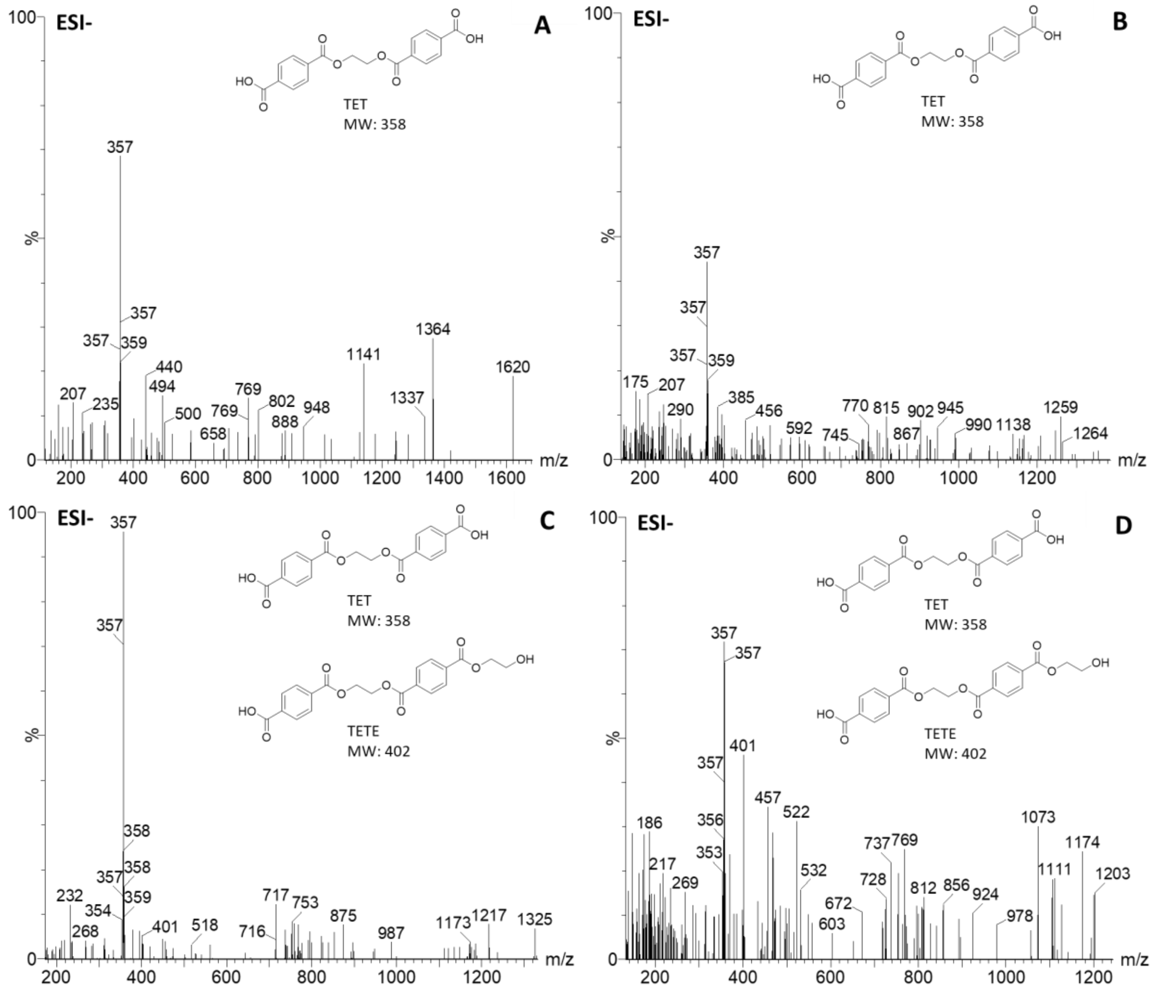

Figure S4. UHPLC-MS spectra of the produced dimer generated by CysP$_m$, OmpA$_m$, EfeO$_m$, and FliD$_m$ after nPET degradation, related to Table 1, Figure S3, and Table S4

(A) UHPLC-MS spectra of degradation-derived dimeric products released from nPET by CysP$_m$

(B) UHPLC-MS spectra of degradation-derived dimeric products released from nPET by OmpA$_m$

(C) UHPLC-MS spectra of released dimeric degradation products from nPET by EfeO$_m$

(D) UHPLC-MS spectra of released dimeric degradation products from nPET by FliD$_m$

(A-D) Samples were analyzed by UHPLC-MS as detailed in STAR★METHODS. The main products from the UHPLC analysis of each sample ionized at m/z 357 ($C_{18}H_{14}O_8$) and m/z 401 ($C_{20}H_{18}O_9$). These findings are consistent with the products being TET and TETE. All other original data were generated by the Unidad de Análisis Instrumental, Instituto de Química Médica (IQM-CSIC), Spain. Raw data (Data S2B – mass spectrometry files compatible with MassLynx V4.1) can be provided upon request by the LC–MS unit. The analysis shows that with CysP$_m$ and OmpA$_m$, TET was detected among the degradation products, whereas with EfeO$_m$ and FliD$_m$, both TET and TETE were present. In Figure 4, Table 1, Figure S3, and Table S4, these products are collectively referred to as “others”.

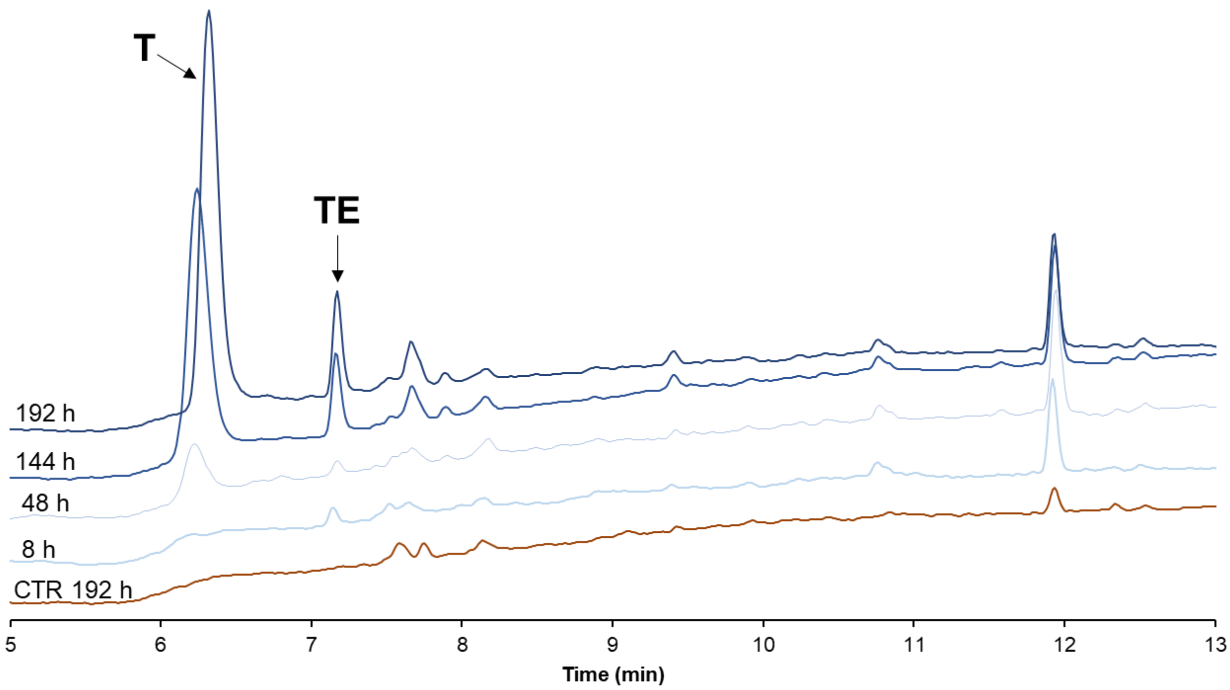

Figure S5. Time-course HPLC chromatograms recorded at 242 nm of PET degradation products released by engineered *E. coli* S3 strain grown on PET powder

HPLC chromatograms of culture supernatants from the engineered *E. coli* strain incubated with pPET in M9 medium at 37°C, sampled at different time points (8-192 h). The main degradation products, identified by comparison with retention time standards, were T and TE. Additional peaks appeared throughout the chromatograms, especially at later retention times, consistent with larger or structurally distinct oligomers. These compounds were not quantified due to the lack of standards, indicating that total PET degradation shown in Figure 4A, may exceed the 97 µM measured for T and TE alone. The control (CTR) corresponds to a 192 h culture of strain S1 expressing only engineered $LsrB_m$.

Table S1. Capacity of published native and engineered microorganisms to hydrolyze PET

The table summarizes the concentrations of PET hydrolysis products obtained from different PET forms, including films, powders, and particles of various sizes, by selected native and engineered microorganisms reported in the literature. This list is not exhaustive, but rather aims to highlight representative strains from diverse taxonomic groups and the synthetic biology tools employed in their design. Data are drawn from previously published studies (see references).

Strain	PET degrading system	Engineering system	PET type	[PET]	Medium	T (°C)	Time (h)	[T]+[TE] (μM)	Ref.
Ideonella sakaiensis[1,7]	Secreted *Is*PETase and *Is*METHase	-	PET film	n.d.	YSV medium	30	18	~300	[S1]
Clostridium thermocellum DSM1313::pHK-LCC[2,7]	Secreted LCC	Plasmid pHK	Goodfellow Ltd. ES301445 amorphous PET film	~50 mg / 10 mL	GS-2 medium with 5 g / L cellobiose	60	336	12900	[S2]
Clostridium thermocellum DSM1313::pHK-LCC[2,7]	Secreted LCC	Plasmid pHK	Goodfellow Ltd. ES301445 amorphous PET film	~50 mg / 10 mL	GS-2 medium with 5 g / L of cellobiose	60	240	~5000	[S3]
Clostridium thermocellum DSM1313::pHK-LCC[2,8]	Secreted LCC	Plasmid pHK	Post-consumer heated PET particles from Coca-Cola bottles (~0.9 mm)	2500 mg / 1000 mL	GS-2 medium, with 2 % (w/v) Avicel	60	240	~20000-25000	[S3]
Clostridium thermocellum DSM1313::pHK-LCC_I[2,7]	Secreted LCC_I (F243I)	Plasmid pHK	Goodfellow Ltd. ES301445 amorphous PET film	~50 mg / 10 mL	GS-2 medium with 5 g / L of cellobiose	60	240	~15000	[S3]
Clostridium thermocellum DSM1313::pHK-LCC_HFB7[2,7]	Secreted chimera LCC-HFB7	Plasmid pHK	Goodfellow Ltd. ES301445 amorphous PET film	~50 mg / 10 mL	GS-2 medium with 5 g / L of cellobiose	60	240	~12000	[S3]
Clostridium thermocellum Δ*pyrF*::P2638-LCC-HFB7[2,8]	Secreted chimera LCC-HFB7[3]	Genome *knock-in*	Post-consumer PET particles from Coca-Cola bottles (~0.9 mm)	2500 mg / 1000 mL	GS-2 medium, with 2 % (w/v) Avicel	60	240	~20000-25000	[S3]
Wastewater bacteria 2 (*Pusillimonas* ssp.)[2,4]	Secreted FAST-PETase	Conjugative plasmid pFAST-PETase-cis	Post-consumer PET particles from coffee cup lid (~0.9 mm)	6 mm / 2 mL	0.1 M KH 2 PO 4 -NaOH buffer pH 8.0	50	120	n.d.	[S4]
Wastewater bacteria 5 (*Enterobacter* ssp.)[2,4]	Secreted FAST-PETase	Conjugative plasmid pFAST-PETase-cis	Post-consumer PET particles from coffee cup lid (~0.9 mm)	6 mm / 2 mL	0.1 M KH 2 PO 4 -NaOH buffer pH 8.0	50	120	n.d.	[S4]

Wastewater bacteria 7 (*Escherichia* ssp.)[2,4]	Secreted FAST-PETase	Conjugative plasmid pFAST-PETase-cis	Post-consumer PET particles from coffee cup lid (~0.9 mm)	6 mm / 2 mL	0.1 M KH 2 PO 4 -NaOH buffer pH 8.0	50	120	n.d.	[S4]
Vibrio natriegens 14048 dns::LacI-T7-RNAP[2,8]	Anchored chimera *Is*PETase-*Is*METHase	Plasmid pUC19	Goodfellow Ltd. ES30-PD-000132 PET powder (<300 µm, >40% crystallinity)	6 mg / 6 mL	M9v2 medium with 0.4% (w/v) glucose	30	168	3	[S5]
Vibrio natriegens 14048 *dns*::LacI-T7-RNAP[2,8]	Anchored chimera FastPETase-*Is*METHase	Plasmid pUC19	Goodfellow Ltd. ES30-PD-000132 PET powder (<300 µm, >40% crystallinity)	6 mg / 6 mL	M9v2 medium with 0.4% (w/v) glucose	30	168	4	[S5]
Bacillus subtillis WB600-P43-SP_{amy}[2,8]	Secreted *Is*PETase[5]	Plasmid pUC19	Dupont PET film (0.0125 mm thick)	1 cm^2 / n.d.	LB medium	28	36	n.d.	[S6]
Bacillus subtillis WB600-P43-SP_{PETase}[2,8]	Secreted *Is*PETase[5]	Plasmid pUC19	Dupont PET film (0.0125 mm thick)	1 cm^2 / n.d.	LB medium	28	36	n.d.	[S6]
Escherichia coli MG1655[2,6]	Secreted LCC	Plasmid pSEVA238	Solvent pre-treated Goodfellow Ltd. ES301445 amorphous PET	10 mg / 1 mL	LB medium	30	504	~600	[S7]
Escherichia coli MG1655[2,6]	Secreted chimera *Is*PETase-*Is*METHase	Plasmid pSEVA238	Solvent pre-treated Goodfellow Ltd. ES301445 amorphous PET	10 mg / 1 mL	LB medium	30	504	~4200	[S7]
Escherichia coli MG1655[2,6]	Secreted PHL7	Plasmid pSEVA238	Solvent pre-treated Goodfellow Ltd. ES301445 amorphous PET	10 mg / 1 mL	LB medium	30	504	~3800	[S7]
Escherichia coli MG1655[2,6]	Secreted FAST-PETase	Plasmid pSEVA238	Solvent pre-treated Goodfellow Ltd. ES301445 amorphous PET	10 mg / 1 mL	LB medium	30	504	~4000	[S7]
Stenotrophomonas pavanii G1-PET[2,8]	Secreted DuraPETase	Plasmid pBBR1-P24-SP1	Post-consumer PET particles from Wahaha bottle	50 mg / 60 mL	YS buffer	30	168	11.03	[S8]

			(~0.5 mm, ~32% crystallinity)						
Stenotrophomonas pavanii G1-ICCG[2,8]	Secreted LCC-ICCG	Plasmid pBBR1-Ptac	Post-consumer PET particles from Wahaha bottle (~0.5 mm, ~32% crystallinity)	50 mg / 60 mL	YS buffer	30	168	9.36	[S8]
Escherichia coli PHL628[2,8]	Curli bonded *Is*PETase	Plasmid pBbE1a-CsgA	Goodfellow Ltd. ES30-PD-000132 PET powder (<300 µm, >40% crystallinity)	20 mg / 1.5 mL	50 mM glycine-NaOH buffer pH 9	30	168	~3250	[S9]
Escherichia coli PHL628[2,8]	Curli bonded *Is*PETase	Plasmid pBbE1a-CsgA	Goodfellow Ltd. ES30-PD-000132 PET powder (<300 µm, >40% crystallinity)	20 mg / 1.5 mL	50 mM glycine-NaOH buffer pH 9	37	168	~2000	[S9]
Yarrowia lipolytica ADJ 2 pAD PET_IS[2,7]	Secreted *Is*PETase	Plasmid pAD	Goodfellow Ltd. ES30-PD-000132 PET powder (<300 µm, >40% crystallinity)	2000 mg / 30 mL	YPD medium with 50 g / L glucose	28	240	3190	[S10]
Phaeodactylum tricornutum[5,9]	Secreted $PETase^{R280A}$	pPha-NR shuttle vector	Shredded PET from ALPLA-Werke Lehner GmbH & Co KG (Gemünden, Germany)	10 g / 150 mL	f/2-medium with NH_4^+	30	168	n.d.	[S11]
Rhodococcus pyridinivorans P23[1,5,8]	Transmembrane PET esterase (OQN32_06240)	-	Goodfellow Ltd. PET film GF89357619	1 cm^2 / 0.3 mL	300 mM Na_2HPO_4 - NaH_2PO_4	30	60	1.5	[S12]
E. coli BL21 (DE3) Strain S1[2,7]	Periplasmatic $LsrB_m$ + FucO + AldA	Genome *knock-in* + plasmid pET-45b(+)	Goodfellow Ltd. ES301445 amorphous PET particles (0.1-5.5 nm, 1.3% crystallinity)	13 mg / 10 mL	M9 medium with 0.1 g / L glycerol	37	22	7263 ± 215	[S13]
E. coli BL21 (DE3) Strain S3[2,7]	Outer membrane bound $OmpA_m$ + Periplasmatic $LsrB_m$ + FucO + AldA	Genome *knock-in* + plasmid pET-45b(+)	Goodfellow Ltd. ES30-PD-000132 PET powder (<300 µm, >40% crystallinity)	15 mg / 0.5 mL	M9 medium with 0.1 g / L glycerol	37	24	157 ± 2	This study

[1]Native strain. [2]Engineered strain. [3]Controlled pH**.** [4]Culture supernatants, only absorbance 240 nm (no HPLC or UHPLC). [5]Microscopy. [6]Culture supernatants. [7]Culture growth. [8]Resting cells. [9]Microalga.

Table S4. Specific activities of engineered variants $CysP_m$, $OmpA_m$, $EfeO_m$, and $FliD_m$ during pPET and nPET hydrolysis at 40 and 60°C, compared with previously reported variants $SsuA_m$ and $LsrB_m$

Variant	Mutation	Active site	PET hydrolysis (μmol h^{-1} g^{-1})			
			nPET		pPET	
			40°C	60°C	40°C	60°C
$CysP_m$[a]	Q72H, S31D	S65, H72, D31	148 ± 6	1876 ± 14	11 ± 10	95 ± 2
$OmpA_m$[a]	A92S, R81H, K94E	S92, H81, E94	245 ± 5	1778 ± 13	46 ± 6	115 ± 3
$EfeO_m$[a]	A199S, Y204H	S199, H204, D210	0	746 ± 6	0	18 ± 0
$FliD_m$[a]	N260S, E240H, N253E	S365, H338, D372	108 ± 9	1741 ± 17	0	95 ± 3
$SsuA_m$[b]	A65S, P67H, P87D	S65, H67, D87	988 ± 0	1167 ± 4	340 ± 40	2740 ± 110
$LsrB_m$[b]	G318S, D317H, S287D	S318, H317, D287	2401 ± 60	1936 ± 14	1090 ± 100	19620 ± 590

[a]Reaction conditions: [pPET], 7 mg mL^{-1} or [nPET], 1.65 mg mL^{-1}; [enzyme], 0.1 mg mL^{-1}; buffer, 50 μL of 40 mM HEPES buffer; pH, 7.0; agitation, 950 rpm. The reaction times were set to 0, 2, 4 and 24 h (only 24 h data were used for calculations). Analyses were performed by HPLC. All values represent the means of three independent biological replicates (n = 3), with standard deviations calculated using the STDEV.S function in Excel 2024. Unprocessed data are available in Data S1B-S1C.

[b]Values from Vidal et al. [S13]. Reaction conditions: [pPET], 7 mg mL^{-1} or [nPET], 1.65 mg mL^{-1}; [enzyme], 0.1 mg mL^{-1}; buffer, 50 μL of 40 mM HEPES buffer; pH, 7.0; agitation, 950 rpm.

Table S5. Degradation products identified and confirmed by HPLC (using standards) and UHPLC-MS for *E. coli* BL21(DE3) proteins engineered to display PETase activity

Enzyme	T[2]	TE[2]	ETE[2]	TET[2]	TETE[2]
CysP$_m$	✓	✓	✓	✓	X
OmpA$_m$	✓	✓	✓	✓	X
EfeO$_m$	✓	✓	✓	✓	✓
FliD$_m$	✓	✓	✓	✓	✓
SsuA$_m$[1]	✓	✓	✓	✓	✓
LsrB$_m$[1]	✓	✓	✓	✓	✓
Structure	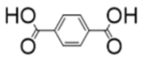	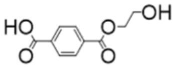	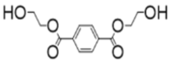	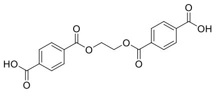	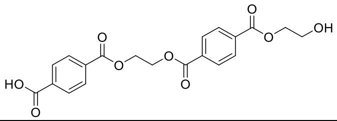

[1]SsuA$_m$ and LsrB$_m$ information extracted from Vidal et al. [S13].
[2]Nomenclature according to Schubert et al. [S14].

Supplemental References

S1. Yoshida, S., Hiraga, K., Takehana, T., Taniguchi, I., Yamaji, H., Maeda, Y., Toyohara, K., Miyamoto, K., Kimura, Y., and Oda, K. (2016). A bacterium that degrades and assimilates poly(ethylene terephthalate). *Science* 351, 1196-1199. https://doi.org/10.1126/science.aad6359.

S2. Yan, F., Wei, R., Cui, Q., Bornscheuer, U.T., and Liu, Y.-J. (2021). Thermophilic whole-cell degradation of polyethylene terephthalate using engineered *Clostridium thermocellum*. *Microb. Biotechnol.* 14, 374-385. https://doi.org/10.1111/1751-7915.13580.

S3. Liu, Y.-J., Yan, F., Dong, W., Sun, Y., Wei, R., and Feng, Y. (2025). Optimized whole-cell depolymerization of polyethylene terephthalate to monomers using engineered *Clostridium thermocellum*. *J. Hazard. Mater.* 488, 137441. https://doi.org/10.1016/j.jhazmat.2025.137441.

S4. Yip, A., McArthur, O.D., Ho, K.C., Aucoin, M.G., and Ingalls, B.P. (2024). Degradation of polyethylene terephthalate (PET) plastics by wastewater bacteria engineered via conjugation. *Microb. Biotechnol.* 17, e70015. https://doi.org/10.1111/1751-7915.70015.

S5. Li, T., Menegatti, S., and Crook, N. (2023). Breakdown of polyethylene terephthalate microplastics under saltwater conditions using engineered *Vibrio natriegens*. *AIChE J.* 69, e18228. https://doi.org/10.1002/aic.18228.

S6. Wang, N., Guan, F., Lv, X., Han, D., Zhang, Y., Wu, N., Xia, X., and Tian, J. (2020). Enhancing secretion of polyethylene terephthalate hydrolase PETase in *Bacillus subtilis* WB600 mediated by the SPamy signal peptide. *Lett. Appl. Microbiol.* 71, 235-241. https://doi.org/10.1111/lam.13312.

S7. Banks, A.M., Abdulmutalib, U., Sonnendecker, C., Kim, J., Bosomworth, C., Brown, S., Wei, R., Álvarez-Ortega, C., Pomposiello, P., Avignone-Rossa, C., Larrouy-Maumus, G., Zimmermann, W., and Jiménez, J.I. (2024). Degradation of PET plastic with engineered environmental bacteria. Preprint at *bioRxiv*. https://doi.org/10.1101/2024.09.24.614569.

S8. Huang, Q.-S., Chen, S.-Q., Zhao, X.-M., Song, L.-J., Deng, Y.-M., Xu, K.-W., Yan, Z.-F., and Wu, J. (2024). Enhanced degradation of polyethylene terephthalate (PET) microplastics by an engineered *Stenotrophomonas pavanii* in the presence of biofilm. *Sci. Total Environ.* 955, 177129. https://doi.org/10.1016/j.scitotenv.2024.177129.

S9. Zhu, B., Ye, Q., Seo, Y., and Wei, N. (2022). Enzymatic degradation of polyethylene terephthalate plastics by bacterial curli display PETase. Environ. *Sci. Technol. Lett.* 9, 650-657. https://doi.org/10.1021/acs.estlett.2c00332.

S10. Kosiorowska, K.E., Moreno, A.D., Iglesias, R., Leluk, K., and Mirończuk, A.M. (2022). Production of PETase by engineered *Yarrowia lipolytica* for efficient poly(ethylene terephthalate) biodegradation. *Sci. Total Environ.* 846, 157358. https://doi.org/10.1016/j.scitotenv.2022.157358.

S11. Moog, D., Schmitt, J., Senger, J., Zarzycki, J., Rexer, K.-H., Linne, U., Erb, T., and Maier, U.G. (2019). Using a marine microalga as a chassis for polyethylene terephthalate (PET) degradation. *Microb. Cell Fact.* 18, 171. https://doi.org/10.1186/s12934-019-1220-z.

S12. Guo, W., Duan, J., Shi, Z., Yu, X., and Shao, Z. (2023). Biodegradation of PET by the membrane-anchored PET esterase from the marine bacterium *Rhodococcus pyridinivorans* P23. *Commun. Biol.* 6, 1090. https://doi.org/10.1038/s42003-023-05470-1.

S13. Vidal, P., Giménez-Dejoz, J., Fernandez-Lopez, L., Romero, S., Nazemi, S.A., Luengo, M., Gonzalez-Alfonso, J.L., Martinez-Sugrañes, M., Robles-Martín, A., Almendral, D., et al. (2025). Computationally guided genome rewiring of *Escherichia coli* and its application for nanopolyethylene terephthalate (PET) biodegradation and upcycling. *Trends Biotechnol.* 43, 2874-2903 https://doi.org/10.1016/j.tibtech.2025.07.008.

S14. Schubert, S., Schaller, K., Arnling Bååth, J., Hunt, C., Borch, K., Jensen, K., Brask, J., and Westh, P. (2023). Reaction pathways for the enzymatic degradation of poly(ethylene terephthalate): What characterizes an efficient PET-hydrolase? *ChemBioChem* 24, e202200516. https://doi.org/10.1002/cbic.202200516.
